# CO_2_ Capture: A Comprehensive Review and Bibliometric Analysis of Scalable Materials and Sustainable Solutions

**DOI:** 10.3390/molecules30030563

**Published:** 2025-01-26

**Authors:** Domingo Cesar Carrascal-Hernández, Carlos David Grande-Tovar, Maximiliano Mendez-Lopez, Daniel Insuasty, Samira García-Freites, Marco Sanjuan, Edgar Márquez

**Affiliations:** 1 Departamento de Química y Biología, Facultad de Ciencias Básicas, Universidad del Norte, Barranquilla 080020, Colombia; domingoh@uninorte.edu.co (D.C.C.-H.); maximilianom@uninorte.edu.co (M.M.-L.); insuastyd@uninorte.edu.co (D.I.); 2 Grupo de Investigación de Fotoquímica y Fotobiología, Programa de Química, Universidad del Atlántico, Carrera 30 No 8–49, Puerto Colombia 081007, Colombia; 3 Centro de Investigación e Innovación en Energía y Gas—CIIEG, Promigas S.A. E.S.P., Barranquilla 11001, Colombia; samira.garcia@promigas.com (S.G.-F.); marco.sanjuan@promigas.com (M.S.)

**Keywords:** carbon capture, utilization and storage, absorption, adsorption, climate change, CO_2_

## Abstract

The greenhouse effect and global warming, driven by the accumulation of pollutants, such as sulfur oxides (SOx), nitrogen oxides (NOx), and CO_2_, are primarily caused by the combustion of fossil fuels and volcanic eruptions. These phenomena represent an international crisis that negatively impacts human health and the environment. Several studies have reported novel carbon capture, utilization, and storage (CCUS) technologies, promising solutions. Notable methods include chemical absorption using solvents, and the development of functionalized porous materials, such as MCM-41, impregnated with amines like polyethyleneimine. These technologies have demonstrated high capture capacity and thermal stability; however, they face challenges related to recyclability and high operating costs. In parallel, biodegradable polymers and hydrogels present sustainable alternatives with a lower environmental impact, although their industrial scalability remains limited. This review comprehensively analyzes CO_2_ capture methods, focusing on silica-based porous supports, polymers, hydrogels, and emerging techniques, like CCUS and MOFs, while including traditional methods and a bibliometric analysis to update the field’s scientific dynamics. With increasing investigations focused on developing new CCUS technologies, this study highlights a growing interest in eco-friendly alternatives. A bibliometric analysis of 903 articles published between 2010 and 2024 provides an overview of current research on environmentally friendly carbon capture technologies. Countries such as the United States, the United Kingdom, and India are leading research efforts in this field, emphasizing the importance of scientific collaboration. Despite these advancements, implementing these technologies in industrial sectors with high greenhouse gas emissions remains scarce. This underscores the need for public policies and financing to promote their development and application in these sectors. Future research should prioritize materials with high capture capacity, efficient transformation, and valorization of CO_2_ while promoting circular economy approaches and decarbonizing challenging sectors, such as energy and transportation. Integrating environmentally friendly materials, energy optimization, and sustainable strategies is essential to position these technologies as key tools in the fight against climate change.

## 1. Introduction

CO_2_ is vital for the carbon cycle and is naturally produced through respiration and volcanic eruptions. However, the increase in anthropogenic emissions, primarily from the consumption of fossil fuels in energy production, has made CO_2_ the leading greenhouse gas [1,2,3]. The dependence on fossil fuels has led to a significant increase in global CO_2_ emissions since the Industrial Revolution, with atmospheric concentrations rising from 278 parts per million (ppm) in 1750 to 421.20 ppm in 2024, according to recent reports from the Global Monitoring Laboratory of the National Oceanic and Atmospheric Administration (NOAA) (https://gml.noaa.gov/ccgg/trends/global.html (accessed on 10 September 2024)). In this context, the most relevant anthropogenic greenhouse gas emissions are CO_2_, accounting for 73.7% of the total gases recorded, followed by methane at 18.3%, nitrogen oxides at 5.6%, and, to a lesser extent, fluorinated gases (or those containing fluorine, mainly used in refrigerants) at 2.4% [4]. Among the countries with the highest emissions of these gases are China with 32.5%, the United States with 12.6%, the European Union with 7.3%, India with 6.7%, Russia with 4.7%, and Japan with 3.0%. These emissions are attributed to the energy sector with 36.6%, manufacturing with 21.5%, the transportation industry with 20.1%, construction and metal smelting with 12.1%, and other sectors, to a lesser extent, with 9.4% of total greenhouse gas emissions [4]. This accelerated trend in pollutant emissions has led to a 0.6 °C increase over the past decade. Global temperatures are estimated to rise by 1.5 °C between 2030 and 2050, according to reports from the Intergovernmental Panel on Climate Change (IPCC), which will hurt human health and ecosystems [5].

In light of this situation, several studies have reported carbon capture, utilization, and storage (CCUS) as a promising technology in the fight against climate change, as it offers a multidisciplinary approach to addressing rising CO_2_ emissions and climate change [6,7,8,9]. This technology has the potential to significantly reduce net CO_2_ emissions on a large scale, with applications in power plants, coal, and gas mining, facilitating decarbonization in energy industries [8]. However, CCUS faces significant technical and economic challenges that limit its industrial scalability. One of the main obstacles is the high financial investment required, making it an unprofitable option in the short term. To facilitate its adoption and scalability, it is essential to promote innovation in this technology and establish an economic framework that incentivizes its development, thereby enabling CCUS to become a viable alternative for emissions reduction, as proposed by several published studies [10,11,12,13,14].

Additionally, methods have been developed for synthesizing hydrogels designed for CO_2_ capture [15]. A notable example is the synthesis of polyethyleneimine (PEI)-based hydrogels, which result in a porous polymer matrix capable of reducing CO_2_ concentration in combustion gases with high humidity. This material exhibits remarkable adsorption capacity, reaching up to 4.85 mmol per gram of adsorbent at 40 °C through an endothermic process at room temperature. These advances represent a significant step toward more effective solutions for managing CO_2_ emissions [16]. PEI-based hydrogels for CO_2_ capture have shown good surface area and superior absorption capacity compared to liquid PEI. Furthermore, microwave heating can quickly regenerate this material, allowing recyclability [17]. On the other hand, adding K_2_CO_3_ (a Lewis base) promotes CO_2_ adsorption and transformation within hydrogels prepared from PEI loaded with different amines (primary and secondary). These modifications optimize the formation of carbamates, which can be quickly recovered through endothermic processes [18]. Additionally, the effectiveness of simple systems using non-aqueous amines on solid supports has been investigated, employing cost-effective and accessible materials for direct atmospheric CO_2_ adsorption. For example, hydrogels composed of poly(N-2-hydroxyethylacrylamide) (PHEAA) and polyacrylamide (PAA), impregnated with diethanolamine (DEA) in non-aqueous solvents, such as ethylene glycol, achieved significant capture capacities, reaching up to 5.59%. This performance was further improved using solid support with a larger surface area, allowing adsorption capacities up to 6.37% in these non-aqueous systems. Moreover, these systems exhibit remarkable resistance to high temperatures and repeated cycles, maintaining their effectiveness without losing capacity in the solvent or amine [19].

Similarly, the synthesis of polyacrylic acid beads (AIMG) presents a solid structure that creates spaces between the beads, which is suitable for enhanced diffusion of aqueous amines and facilitates CO_2_ diffusion, improving the interaction between the amines and the gas. This results in higher adsorption efficiency and faster kinetics than conventional aqueous solutions [20]. Additionally, by encapsulating amine solutions in microgels, corrosion issues are significantly reduced, allowing for higher amine concentrations in commercial applications, thanks to their thermally stable network. Furthermore, AIMGs with low-volatility amines have shown favorable cyclic performance during the thermal regeneration process, which is beneficial for scalability [20].

Additionally, it has been demonstrated that poly(N-hydroxyethylacrylamide) hydrogels impregnated with amino acid salts exhibit an absorption capacity of 60 mg of CO_2_ per gram of adsorbent from ambient air. Tests conducted under various conditions have shown that this system is suitable for post-combustion CO_2_ capture, especially when using a polar solvent like ethylene glycol [21]. Hydrogels used for CO_2_ capture share common characteristics, such as the presence of amino groups (R-NH_2_ and R_1_-NH-R_2_). However, in some cases, these matrices may present specific toxicity or may be synthetic, which can lead to accumulation in industrial applications and impact the environment, highlighting the need to explore more sustainable and eco-friendly alternatives for scalability [22].

The three-dimensional crosslinked structure of polymeric materials possesses unique properties, such as expanding upon contact with various solutions, allowing them to retain large amounts of liquid [23]. Their three-dimensional architecture generates pores classified as micropores, mesopores, and macropores, whose dimensions significantly influence adsorption capacity; a higher number of micropores generally results in a larger specific surface area, thus improving CO_2_ capture efficiency [24]. Furthermore, adjusting the degree of crosslinking during synthesis can optimize both the size and distribution of the pores. Additionally, the adaptability of hydrogels to various environmental conditions and their resistance to thermal variations and repeated cycles ensure their long-term functionality [24]. These characteristics offer advantages over conventional porous materials or supports, such as silica supports, which feature a porous structure that facilitates fluid diffusion but lacks functional groups or atoms that react with CO_2_. However, this limitation has been overcome by functionalizing these materials for CO_2_ capture applications. For example, an MCM-41 support infused with 70% PEI has demonstrated up to 215 mg per gram of support, which is promising for its use in local CO_2_ emission sources [25].

Despite the information available on CO_2_ capture methods, this comprehensive review deeply analyzes the most relevant information on CO_2_ capture through technologies, such as silica-based porous supports, polymeric materials, and cross-linked materials, such as hydrogels, as an effective solution for CO_2_ capture and functionalization. In addition, it considers the traditional methods of CO_2_ capture (including adsorption and absorption). It discusses their mechanisms of action, recent research, and emerging methods, such as CCUS, using amino acids, polymer-based mesoporous materials, and metal–organic frameworks (MOFs). This approach guarantees an update on the main methods of CO_2_ capture, including bibliometric analysis that allows an understanding of the current dynamics of scientific information on the subject. Given the significance of the information documented in this study, the aim was to conduct a bibliometric analysis of 903 articles published between 2010 and 2024 to map the current state of research on CO_2_ capture and its industrial scalability. This information is potentially helpful for highly industrialized sectors with high greenhouse gas emissions, which could implement environmental policies to foster collaborative development in overcoming economic limitations and facilitating scalability.

## 2. Methodology

This study analyzed citations and abstracts from scientific journals using databases such as Elsevier’s Scopus and Web of Science (WoS), platforms renowned for their extensive coverage and quality in the scientific field [26,27]. Scopus and WoS are the most important databases, hosting a vast collection of peer-reviewed publications and editorial content across various disciplines. These databases not only facilitate access to up-to-date academic information but allow researchers to stay current with the latest trends in their fields, identify potential collaborators, and assess the impact of their work through bibliometric metrics. The richness of content and rigor in selecting sources make Scopus and WoS invaluable resources for the scientific community [28,29].

### Search Strategy and Selection Criteria

**Figure 1:**
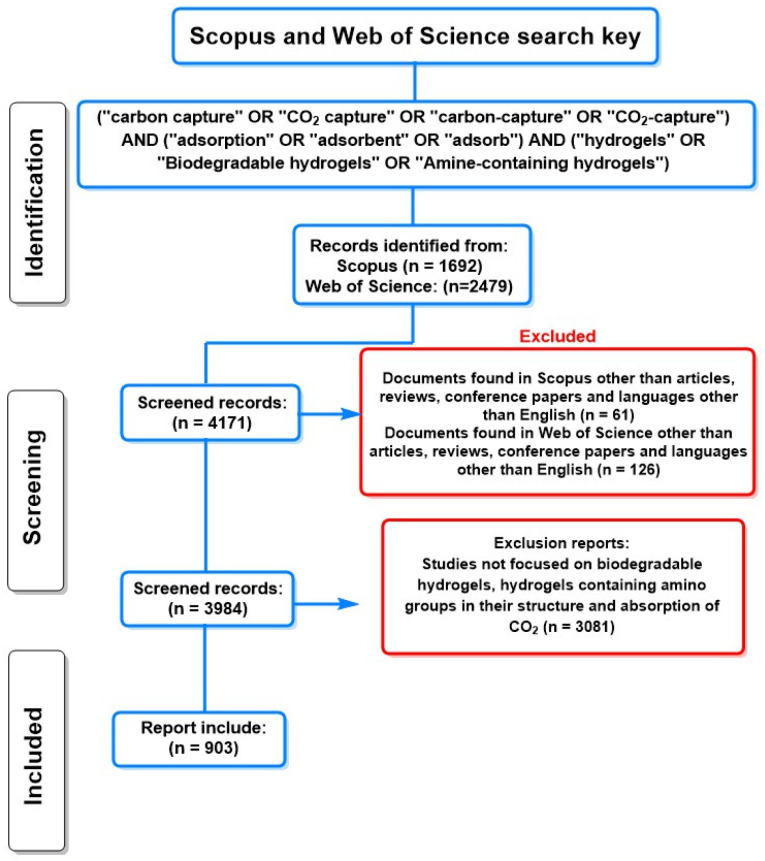
Flowchart of the PRISMA methodology applied to systematic reviews with bibliometric analysis.

The search strategy used in this review article was based on the following query using the PRISMA 2020 parameters [30]: (TS) = TITLE-ABS-KEY (“carbon capture” OR “CO_2_ capture” OR “carbon-capture” OR “CO_2_-capture”) AND (“adsorption” OR “adsorbent” OR “adsorb”) AND (“hydrogels” OR “Biodegradable hydrogels” OR “Amine-containing hydrogels”). This initial search yielded 4171 documents extracted from Scopus and WoS up to September 2024, as illustrated in Figure 1. The search was then refined to focus on specific document types, limiting the results to “articles”, “reviews”, and “conference papers”. Documents published in 2024 were excluded, and the language was restricted to English. Additionally, all titles and keywords unrelated to biodegradable polymers, amine-containing polymers, and CO_2_ adsorption were removed. As a result of this screening process, 990 relevant documents addressing CO_2_ capture through the use of biodegradable hydrogels were identified. This methodical approach ensures that the systematic review is based on the pertinent and updated literature, enabling a more accurate assessment of technologies and methods related to carbon capture.

Finally, to conduct a comprehensive analysis of the most relevant information published on CO_2_ capture and transformation, a detailed review of the results was carried out using VOSviewer [31]. VOSviewer (www.vosviewer.com (accessed on 10 September 2024); Van Eck and Waltman, 2009–2022, version 1.6.18, Leiden University, The Netherlands) is a free-access software designed to create network maps representing institutions, countries, keywords, and citations per article. This program stands out in bibliometric analysis by facilitating the visualization of complex relationships between scientific publications, allowing researchers to identify patterns and trends in the literature [31].

The most relevant data contributing to a deeper understanding of this constantly evolving field were extracted during this process. All collected data were systematically analyzed and are presented in the Literature Review Section on CO_2_ absorption. This section discusses emerging trends in using biodegradable hydrogels and polymeric materials for CO_2_ capture. This study focused on biodegradable hydrogels rather than metal-organic frameworks (MOFs) as eco-friendly technologies for climate change mitigation due to their one-pot synthesis methods, which characterize most hydrogel synthesis processes and are suitable for industrial production. Additionally, this analysis provides a clear overview of the current state of research and identifies key areas for future investigations and practical applications. By understanding the current trends and developments in this area, the goal is to contribute to advancing knowledge on sustainable CO_2_ capture and transformation technologies, which is crucial for addressing contemporary environmental challenges.

## 3. Results and Discussion

### 3.1. How Is Collaboration Between Countries on Technologies for CO_2_ Capture?

Table 1 presents the top ten countries in CO_2_ capture research, highlighting the collaboration between countries in CO_2_ capture studies. These collaborations are crucial as they facilitate the development and scalability of innovative technologies to combat CO_2_ accumulation in highly industrialized sectors. These sectors are important because they sustain economic frameworks in many regions and countries but are problematic due to their high CO_2_ emissions. For example, China leads the list with 323 publications, followed by the United States with 89 documents, India with 79, and Australia with 42. Portugal is in tenth place with five publications. However, it is essential to note that the number of publications per country does not adequately reflect the level of collaboration with other countries in the study of emerging carbon capture technologies. To understand this, it is essential to use correlation maps obtained through VOSviewer, which show the correlation of the trends studied across various nodes and clusters.

**Figure 2:**
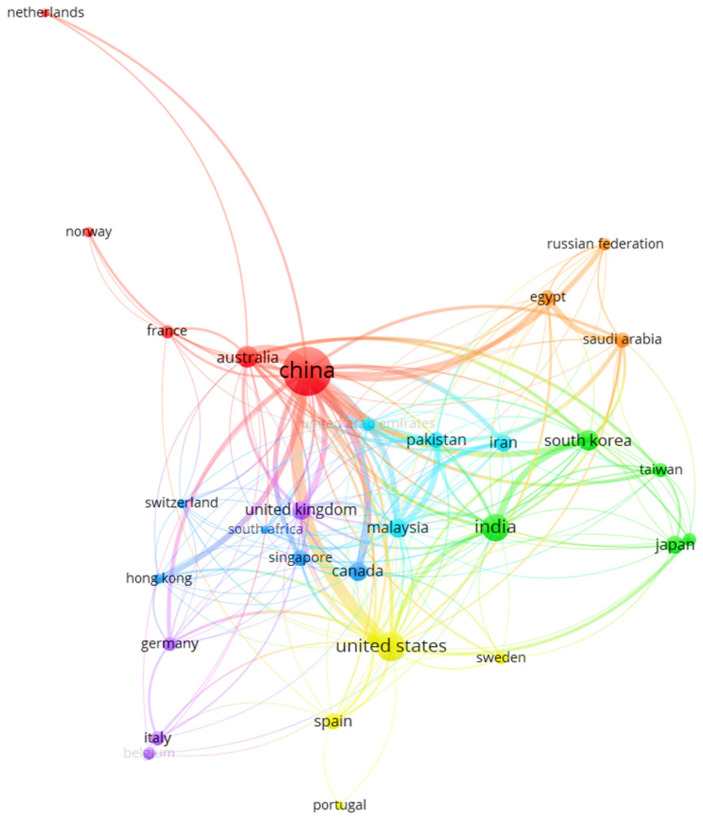
Co-authorship network between countries working on CO_2_ capture technologies (seven clusters). The size of the nodes indicates the number of publications produced by the country. The proximity of two nodes indicates the relationship of their co-authorship link, while the thickness of the connection line indicates the strength of the cooperation. Articles: 31. Links: 211. Total link strength: 466.

In this regard, the correlation map, as shown in Figure 2, illustrates that China, the United States, the United Kingdom, and India have the highest level of global collaboration, as evidenced by seven clusters, reflected in the nodes’ proximity and size. This finding highlights the growing concern about the impact of CO_2_ emissions on the environment, as their accumulation contributes to the greenhouse effect and global warming, phenomena that lead to droughts, intense storms, accelerated glacier melting, and repercussions on public health and ecosystems. The bibliometric map, as shown in Figure 2, illustrates the clusters or nodes represented by colors that facilitate the visual identification of the thematic areas within the analyzed data set. The connecting lines that join the nodes indicate their relationships, such as co-authorships. The thickness of these lines varies, reflecting the strength or relevance of the relationship; thicker lines suggest stronger connections, while thinner lines indicate less significant interactions. In this context, three distinct groups emerge: the first group, represented in red, is led by China, which has 323 publications, a total link strength of 152, and 28 links.

The collaboration between China and Australia stands out with a link strength of 20, followed by an association with the United States, with a link strength of 15. Another significant group is that of the United States, which has a total link strength of 72, supported by 89 publications and 26 links. In particular, the United States represents the most effective global cooperation, thanks to the universality of its language, advanced technologies, and collaborations in various techniques used to scale new CO_2_ capture technologies, despite not having as many documents as China.

The third group is represented by India, which has 79 documents in the field of CO_2_ capture research, with a total link strength of 46 and 19 links. This performance highlights India’s involvement in this crucial area for environmental sustainability. It is worth mentioning that, in addition to the United States, Canada, and Australia, European and Asian countries are the most active collaborators with these leaders in CO_2_ capture research. This international collaboration is essential for addressing global climate change challenges, as it enables the exchange of knowledge, technologies, and best practices between nations. Cooperation among these countries enhances their capabilities and contributes to a collective effort to mitigate the environmental impact of CO_2_ emissions.

### 3.2. In Which Journals Are These Technologies for CO_2_ Capture Being Published?

Table 2 presents the ten most relevant journals in the field of CO_2_ capture research, which is essential for understanding the impact of these studies and the sectors they target. For example, according to the Clarivate Journal Citation Report, these publications cover at least one of the following areas: environmental sciences, environmental chemistry, membrane separation, environmental engineering, capture technologies, chemical engineering, and sustainable green technologies. Notably, except for the journals Industrial & Engineering Chemistry Research and Energy & Fuels, classified as Q2, all the other journals belong to the Q1 category in their respective fields. This highlights the significant impact of “CO_2_ capture technologies” within the scientific community and reveals its application fields, which are directed towards sectors and areas crucial for sustainable development but severely affected by the emission and accumulation of anthropogenic CO_2_.

A notable aspect is the increase in the impact factor of these journals for 2023, according to the Clarivate Journal Citation Reports. This rise indicates a positive trend in consultations and citations, emphasizing the growing importance of using hydrogels for CO_2_ capture as a global concern. Similarly, the 2023 Cite Score, obtained through Scopus for these journals, has also shown growth in recent years, confirming their relevance and the high level of interest they generate among researchers. This context reflects the academic quality of these journals and their fundamental role in disseminating knowledge and innovations necessary to address the environmental challenges related to CO_2_ emissions. Collaboration among researchers and access to these publications are essential for advancing the development of practical solutions in the fight against climate change.

**Figure 3:**
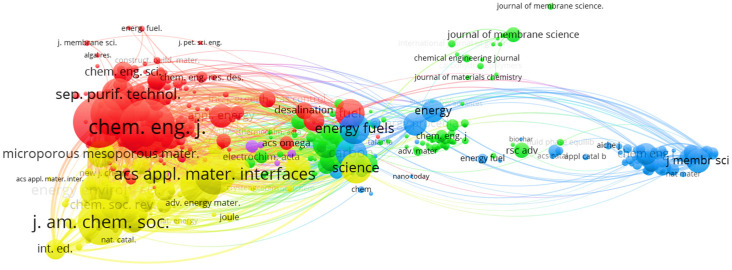
Co-citation network diagram of journals from articles cited at least five times. Articles: 423. Clusters: five. Links: 48,633. Total link strength: 1,995,157.

Figure 3, created with VOSviewer [31], presents a co-citation map between various scientific journals. In this map, the thickness of the lines connecting the nodes (representing journals) indicates the level of co-citation between them. The size of each node reflects the overall strength of the journal, while the thickness of the lines connecting the nodes represents the intensity of co-citation. Similarly, the colors representing the nodes or clusters in the analyzed data indicate the journals that publish similar studies, scope, and fields. As can be seen, the Chemical Engineering Journal and ACS Applied Materials & Interfaces show strong co-citation and notable proximity in the map, suggesting a close thematic relationship between the two. A similar pattern is observed between the journals Energy & Fuels and Fuel, which also exhibit solid collaboration with the previously mentioned journals. Furthermore, both the Journal of Membrane Science and Science and Langmuir are highly cited, indicating their relevance in the field of study. As shown in Figure 3, the proximity of the nodes and the total link strength reflect the cooperation in research focused on CO_2_ capture.

### 3.3. Most Relevant Keywords in CO_2_ Capture

**Figure 4:**
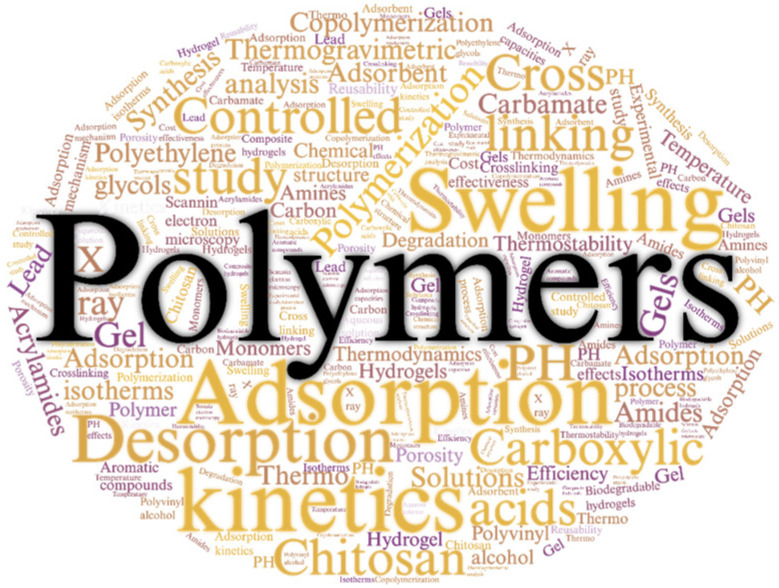
Cloud of the most essential keywords extracted from https://www.nubedepalabras.es/ (accessed on 20 October 2024).

Figure 4 shows the most relevant keywords in this literature review, which has identified key terms in this field, such as “Adsorption”, “Desorption”, “Swelling”, “Kinetics”, “Amines”, “Polymers”, and “Chitosan”, among others. The significance of these terms is closely related to CO_2_ capture and conversion processes, where amines play a crucial role. These chemicals are recognized for their ability to absorb CO_2_, making them key components in climate change mitigation technologies.

**Figure 5:**
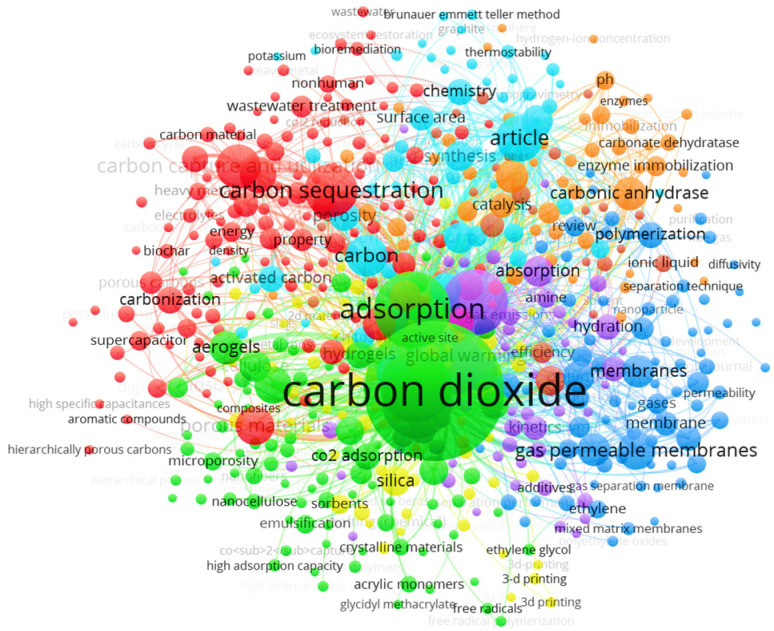
Co-occurrence network based on article weights for terms associated with the first group. The curved lines of varying thickness, representing co-occurrence, illustrate the relationships between the terms. The proximity between the elements reflects the strength of their connection, while the size of each term is determined by its frequency of occurrence: Keywords: 561. Clusters: eight. Links: 31,583. Total link strength: 58,053.

The keyword network was created from terms cited at least ten times, covering 561 words organized into eight groups, with a total link strength of 58,053 (Figure 5). This set of words was obtained after eliminating overly generic terms such as “human”, “animal”, and “non-human”, among others. The first group, represented in green, is related to the impact of CO_2_ on climate change. Within this group, terms such as “Hydrogels”, “Aerogels”, “Kinetics”, and “Porous materials” stand out, with “Carbon dioxide” being the central term, having 556 links, a total link strength of 5813 and 488 occurrences. The second group, in red, is related to CO_2_ capture, storage, and utilization (CCUS), technologies considered essential in the fight against CO_2_ emissions. Within this group, terms like “Carbon sequestration” (with 390 links, a total link strength of 1205, and 97 occurrences) and “Carbon capture and utilization” (with 278 links, a total link strength of 846, and 72 occurrences) stand out. This underscores the importance of recognizing these concepts in the most cited articles globally, reflecting a growing concern about the greenhouse effect.

The third group, represented in light blue, is closely related to publications on CO_2_ adsorption. This group includes terms such as “Thermostability”, “Controlled study”, “Chemistry”, “Gases”, “Surface area”, and “Synthesis”, which reflect solutions to this climate change-related challenge. In this context, terms related to the characterization, synthesis, and analysis of polymers stand out. One notable term is “Gas permeable membrane”, found in the fourth group (in dark blue), with 268 links, a total link strength of 819, and 56 occurrences. The group, represented in orange, focuses on the characteristics and conditions of polymers. Terms such as “pH”, “Scanning electron microscopy”, “Catalysis”, and “Catalytic activity” reflect this focus. The last group, represented in yellow, contains relevant terms such as “Silica”, “3-D printing”, and “Ethylene glycol”, among others, demonstrating the multidisciplinary nature of developing new technologies for CO_2_ capture.

### 3.4. Most Cited Articles on CO_2_ Capture

Table 3 presents the most relevant studies on CO_2_ capture, a global issue that significantly impacts ecosystem stability and threatens public health. Among the most cited articles included in Table 3 are “Direct Capture of CO_2_ from Ambient Air”, with 1580 citations [32], “Separation and Capture of CO_2_ from Large Stationary Sources and Sequestration in Geological Formations—Coalbeds and Deep Saline Aquifers”, “Recent advances in aerogels for environmental remediation applications: A review”, with 551 citations [33], and “A review of the hydrate-based gas separation (HBGS) process for carbon dioxide pre-combustion capture”, with 510 citations [34].

**Figure 6:**
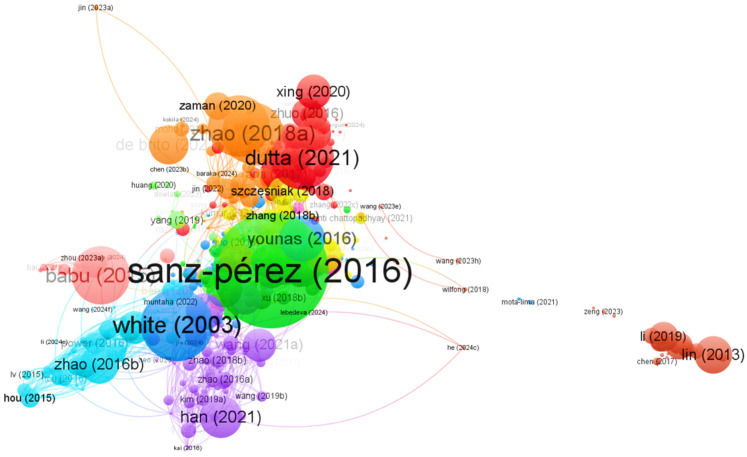
Visualization map of the co-citation network based on the most cited references. The occurrence determines the size of the term; its relationship determines the distance between the elements. Articles: 694. Links: 227. Clusters: 13. Total strength: 4197.

Figure 6 presents the co-citation map of sources that have received more than ten citations. This map highlights the significant relevance of research focused on CO_2_ capture, as reflected in the high co-citation rate, indicated by the thickness of the links and the proximity between the sources. This proximity emphasizes the growing global need for more standardized methodologies and techniques that facilitate the scalability of these technologies.

The bibliometric analysis, which is based on a process that requires the systematic collection and analysis of data related to a specific topic to map the existing academic literature, allowed for the quantitative evaluation and prediction of patterns and trends in research topics, facilitating the identification of emerging areas, an assessment of the impact of publications, and an understanding of the evolution of knowledge. Through statistical tools and techniques, a bibliometric analysis provides a comprehensive view of scientific production, collaborations between authors, and citation networks, as demonstrated by various relevant publications [42,43,44,45].

This bibliometric analysis, which covered 990 relevant documents, indicated that CO_2_ capture is a high impact, constantly expanding field of study. Recent studies have explored various CO_2_ capture, storage, and utilization technologies. This growing interest is driven by the urgency to address climate change and the need to develop sustainable solutions to reduce greenhouse gas emissions. The research encompasses a wide range of approaches, from innovative direct air capture methods to advanced CO_2_ storage and conversion techniques into value-added products, thus reflecting the diversity and complexity of the challenges associated with carbon management in the current context [46,47,48,49,50].

### 3.5. Literature Review: Emerging Technologies in CO_2_ Capture

#### 3.5.1. CO_2_ Capture Methods

Methods for CO_2_ capture have advanced significantly, with various innovative techniques developed to mitigate climate change by reducing CO_2_ concentration in the atmosphere. Adsorption and absorption techniques stand out due to their different mechanisms and applications [51].

Adsorption is based on the adherence of CO_2_ molecules to the surfaces of solid materials, such as activated carbon [52], zeolites [53], and MOFs [54], through weak molecular interactions known as physisorption. This method is particularly effective due to its high selectivity, allowing for efficient separation of CO_2_ in gas mixtures [55]. Recent advancements in nanomaterials and hybrid adsorbents have further increased the efficiency of these processes, making them more viable for industrial applications. Additionally, using renewable energy sources to regenerate the adsorbents represents a sustainable approach to carbon capture [56].

On the other hand, absorption techniques, especially those using liquid solvents, have seen notable innovations [57]. This method allows for the transformation of CO_2_ into valuable compounds, such as carbamates, through a process known as chemisorption, where new covalent bonds are formed. Traditional methods, such as amine absorption, have evolved thanks to introducing advanced solvents that improve CO_2_ solubility and reaction kinetics [58]. A prominent example is phase change solvents, which simplify the separation and capture processes by facilitating transitions between physical states [59].

**Figure 7:**
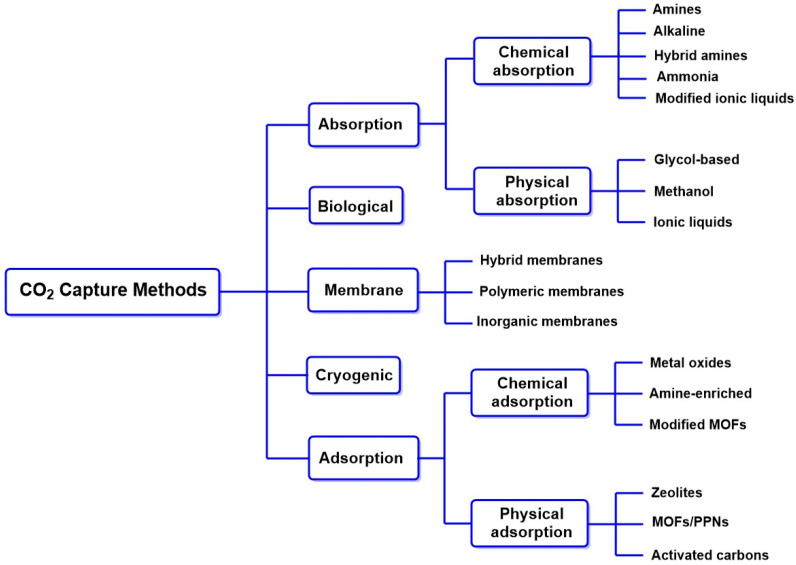
CO_2_ capture methods used in the optimization of technologies based on the capture of anthropogenic CO_2_ [63].

Combining these advanced techniques and the sustainable use of energy resources contributes to reducing global emissions. It offers opportunities to create valuable products from captured CO_2_, thus opening a new era in the fight against climate change. Figure 7 summarizes several CO_2_ capture methods, including adsorption and absorption methods, the use of membranes, biological capture through microalgae, and cryogenic capture. Among these methods, alkaline absorption methods that use solutions, such as NaOH, to generate carbonate/bicarbonate are especially relevant. These products can react with magnesium-rich brines to generate nesquehonite (MgCO_3_∙2H_2_O), magnesium carbonate trihydrate (MgCO_3_∙3H_2_O), and hydromagnesite (Mg_5_(CO_3_)_4_(OH)_2_∙4H_2_O), which have been used as precursors to gypsum-like construction products [60]. In addition, CO_2_ valorization can also occur through its capture with residual glycerol, a residual product that increases exponentially every year in the biodiesel industry. These residual products (glycerol and CO_2_) can generate bio-based copolymers from glycerol carbonate through glycerol carbonate-based vinyl monomers that are incorporated into amphiphilic block copolymers by reversible addition-fragmentation chain transfer (RAFT) polymerization. However, this process presents high production costs, which is a challenge to overcome for its scalability [61].

Adsorption is also a prominent approach in the industry because it can selectively adsorb residual CO_2_ from high-emission local sources (such as using activated carbon in the oil industry). In addition, by adsorbing CO_2_ through weak interactions (physisorption), these materials can release this gas in a controlled manner for its use, allowing the material to be recycled. However, there are still problems to overcome, such as its thermal stability, adsorption capacities, mechanical behavior, and biodegradability [62].

##### Traditional Methods of CO_2_ Absorption/Adsorption

The first gas absorption technology for acidic gases using chemical solvents, specifically alkanol amines, was introduced by Bottoms in 1930. This technology consisted of a triethanolamine (TEA) solution [64]. The mechanism of CO_2_ capture through TEA (a tertiary amine, R_3_-N) consists of two main stages: in the first stage, the solubility and hydration of CO_2_ in the system occur, as shown in Equation (1), which is a slow process. In the second stage, bicarbonate (NaHCO_3_) is formed with the hydroxide ion (-OH). as shown in Equation (2), which is a rapid stage, and enhances mass transfer even at low concentrations of -OH. Subsequently, molecular recognition occurs between the reactants (R_3_-N and CO_2_ in aqueous solution), leading to the reaction, as described in Equation (3). In this reaction, R_3_-N follows a mechanism determined by base-catalyzed hydration, as shown in Equation (3). It has been determined that the reaction kinetics between R_3_-N and CO_2_ is a pseudo-first order reaction. Additionally, CO_2_ capture through R_3_-N is also possible in non-aqueous media, such as ethanol (a polar solvent with high CO_2_ solubility) and chloroform (a low-polarity solvent with moderate CO_2_ solubility); in this context, dissolved CO_2_ will react with solvate R_3_-N to form a pair of ions, which also follows pseudo-first order kinetics, as shown in Equation (2) [65].

(1) $${CO}_{2}+H_{2}O↔{HCO}_{3}^{-}+H^{+}$$

(2) $${CO}_{2}+{OH}^{-}↔{HCO}_{3}^{-}\;$$

(3) $${CO}_{2}+R_{3}N+H_{2}O↔R_{3}{NH}^{+}+{HCO}_{3}^{-}$$

**Figure 8:**
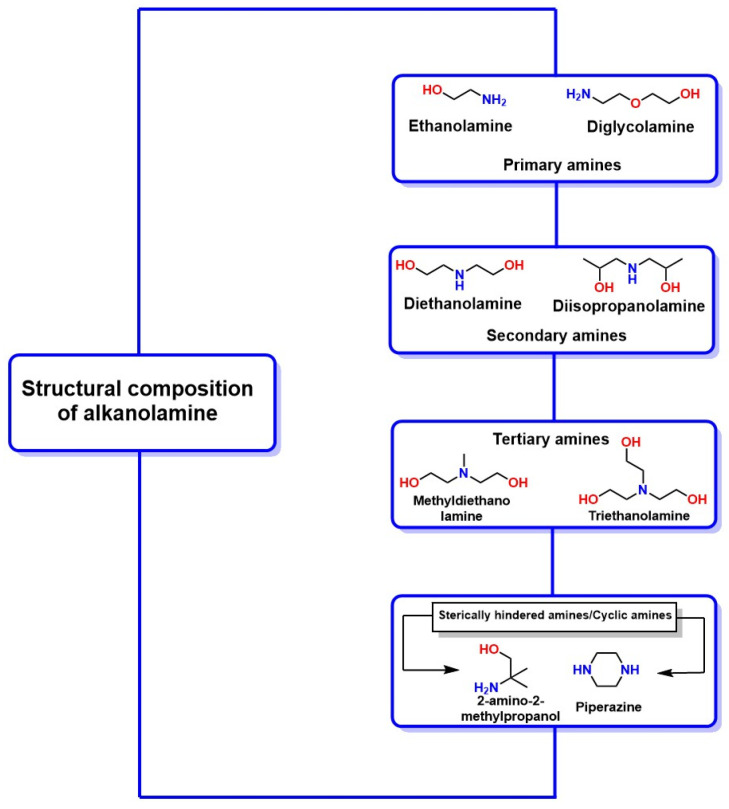
Chemical structure of amines used in acid gas absorption techniques [69,70,71].

These capture systems were used in pilot plants for the preliminary treatment of pollutant gases (Patent code: C10K1/16). In this context, this technology evolved to investigate amines with specific characteristics. For example, it was demonstrated that the presence of -OH groups reduces vapor pressure and improves water solubility (a practical, abundant, and economical solvent), while -NH_2_, -NH-R_1_, and R_1_-N-R_2_ groups enhance basicity in aqueous solutions, favoring the absorption of acidic gases, making this technology attractive for the capture of pollutant gases, such as CO_2_ [66,67]. Figure 8 shows the chemical structure of common alkanolamines that absorb acid gases, such as CO_2_. It highlights the -OH, -NH_2_, NH-R_1_, and R_1_-N-R_2_ groups in their structure, which are essential for enhancing the efficiency and scalability of this technology. In addition to these linear or branched amines, there are cyclic amines, such as piperazine (PZ), which have been used as additives in aqueous solutions of amines, like MEA, DEA, methyl diethanolamine, and 2-amino-2-methylpropanol. The presence of PZ in these solutions optimizes mass transfer and CO_2_ capture by improving the basicity of the solution, thereby favoring the loading of CO_2_. This leads to the reaction between PZ and CO_2_, forming the carbamate ion (PZCOO^−^) and PZ dicarbamate (PZ(COO)_2_), which increases the CO_2_ absorption capacity of the system compared to amine systems without the addition of PZ [68].

Innovative solutions have been developed in the fight against climate change and the reduction of CO_2_ accumulation to advance towards a sustainable future, among which chemical absorption using organic solvents stands out as a key strategy [72]. Due to the specific properties and characteristics of various organic substances, especially alkanolamines, it has been possible to use highly concentrated solutions to enhance their ability to capture and transform CO_2_ from local sources, such as industrial and energy sectors [73]. In this regard, amines, such as diethanolamine (DEA) [74], ethylenediamine (EDA) [75], and monoethanolamine (MEA) [73], have proven effective in CO_2_ capture due to their high reactivity and selectivity. However, despite their effectiveness in CO_2_ capture and transformation, these amines face significant challenges, including toxicity risks from chemical exposure and their corrosive nature, which limits their application in industrial settings [76]. Several studies have highlighted these factors in this context, emphasizing the need for continued research into alternatives and improvements in capture processes to maximize their feasibility and safety in practical applications [77,78,79]. Nonetheless, carbon capture and storage (CCS) has become a proven strategy to reduce the toxicity of these amines, facilitating their scalability to decrease CO_2_ emissions generated by industrial processes and power plants that use fossil fuels [80].

**Figure 9:**
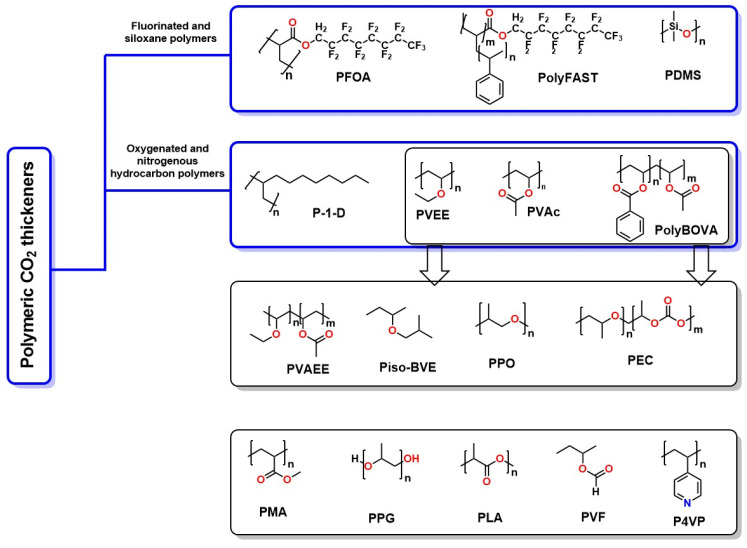
Molecular structures of polymers used as CO_2_ thickeners: PFOA: Poly(1,1-dihydro-perfluorooctyl acrylate) [82]. Polyfast: Poly(fluoroacrylate styrene) [82]. PDMS: Poly(dimethylsiloxane) [83]. P-1D: Poly(1-decane) [84]. PVEE: Poly(vinyl ethyl ether) [85]. PVAc: Poly(vinyl acetate) [86]. PolyBOVA: Poly(benzoyl-vinyl acetate) [86]. PVAEE: Poly(vinyl acetate-vinyl ethyl ether) [87]. Piso-BE: Poly(iso-butyl vinyl ether) [88]. PPO: Poly(propylene oxide) [85]. PEC: Poly(ether carbonate) [89]. PMA: Poly(methyl acrylate) [89]. PPG: Poly(propylene glycol) [89]. PLA: Poly(lactic acid) [89]. PVF: Poly(vinyl formate) [85]. P4VP: Poly(4-vinyl pyridine) [85].

Besides using amines for carbon absorption, other advanced technologies enhance carbon adsorption. Among them, activated carbon adsorption stands out, which utilizes porous materials to efficiently trap CO_2_, and membrane capture, which filters CO_2_ through selective barriers [81]. CO_2_ thickeners, especially polymeric thickeners, play a crucial role in transportation and underground storage by increasing the viscosity of CO_2_ and minimizing leaks, as well as in hydraulic fracturing and oil reservoir exploitation for extraction purposes. These thickeners allow for better fracture propagation and more efficient extraction of trapped oil, improving recovery and reducing environmental impact. Figure 9 shows the chemical structures of various polymers used as CO_2_ thickeners to recover residual oil from oil reservoirs [81]. A fundamental characteristic of these materials is the presence of heteroatoms, such as oxygen, nitrogen, silicon, and halogens, like fluorine; these heteroatoms enhance molecular recognition between the polymer and CO_2_, facilitating retention.

Although these materials have demonstrated good properties for CO_2_ adsorption and moderate mechanical resistance, they present severe limitations. For example, the chemical origin of the monomers that constitute these materials is synthetic, which may be non-biodegradable and exhibit toxicity. This is problematic due to their accumulation, negatively impacting the environment [90].

##### CO_2_ Absorption Through Amino Acids

**Figure 10:**
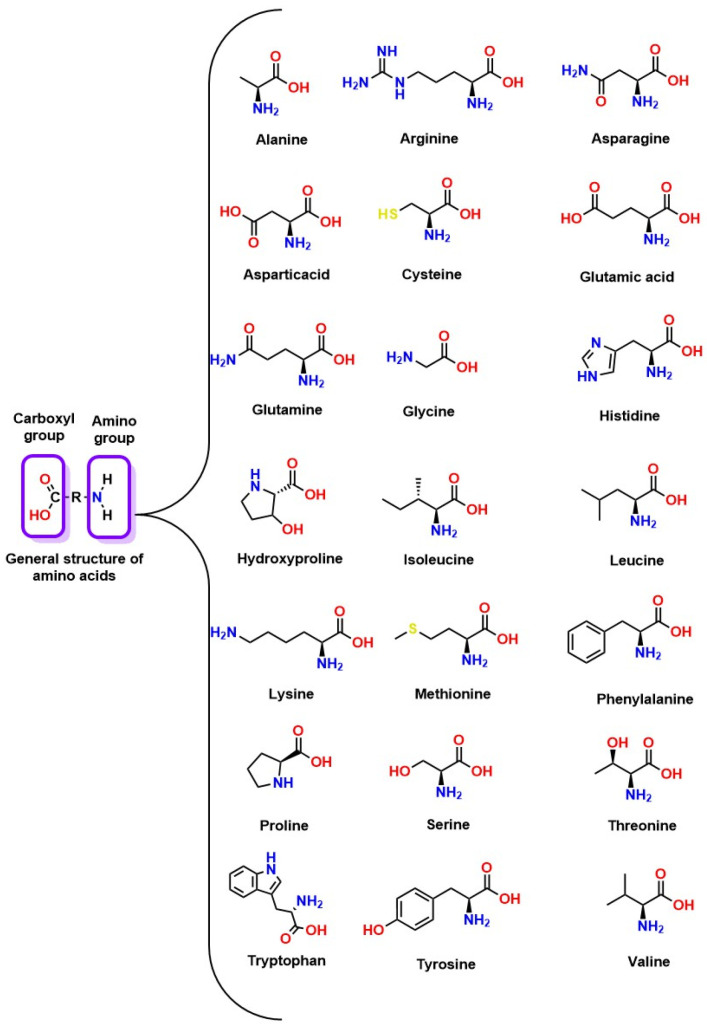
Types of amino acids: their chemical structure contains groups, such as NH_2_, that are reactive with CO_2_, which has attracted attention as potential agents for CO_2_ absorption [91].

Amino acids are organic compounds characterized by the presence of amine (-NH_2_) and carboxyl (-COOH) functional groups, as well as a specific side chain (R group) that varies among different amino acids (as shown in Figure 10). This unique structure has generated a growing interest in their application for CO_2_ absorption. Amino acids can be classified according to various characteristics, such as hydrophobicity and type of enantiomers, allowing a more detailed evaluation of their properties and potential applications in absorption processes [91].

In terms of stereochemistry, amino acids are divided into two categories, D and L, depending on the spatial arrangement of their atoms. This classification is critical as it influences how amino acids interact with other molecules, including CO_2_ [92]. Figure 10 illustrates the general structure of amino acids, providing a visual representation of these essential compounds. Amino acids’ versatility and unique properties make them promising candidates for improving carbon capture and storage technologies.

Aqueous solutions of amino acids can form zwitterions, molecules with positive and negative charges, playing a fundamental role in the reaction between CO_2_ and amino acids [93]. For example, the potassium salts of L-serine and L-proline have been shown to demonstrate absorptions of moles of CO_2_/mol of salt [94]. The basic or deprotonated form of the amino acid acts as the active form in this interaction. For amino acids to react with CO_2_ at neutral pH, they must be deprotonated, which can be facilitated using bases, such as MEA, KOH, and NaOH. Primary amines, such as MEA and DEA, are particularly effective in this context due to their high reactivity, which allows for the formation of products like carbamates, compounds resulting from the reaction of CO_2_ with amines [95].

Two main approaches have been identified regarding the reaction mechanisms of CO_2_ with the amine group: the zwitterion mechanism and the termolecular mechanism. In the zwitterion mechanism, CO_2_ forms a zwitterion before a base removes a proton. On the other hand, the termolecular mechanism involves the simultaneous reaction of an amine with a molecule of CO_2_ and a base [96]. The most commonly used amines in CO_2_ absorption include MEA, DEA, and methyldiethanolamine (MDEA), each showing different adsorption capacities. For example, MEA is known for its efficiency in capturing CO_2_ and its ability to form stable products from this reaction. These findings are crucial for optimizing amino acid-based CO_2_ absorption systems and enhancing their effectiveness in mitigating climate change [97].

In their natural form, the chemical structures of these amino acids exhibit a low reaction rate and a limited capacity to absorb CO_2_ under neutral pH conditions [97]. However, when neutralized with bases, their effectiveness in CO_2_ absorption is significantly improved. Among the counterions used, KOH stands out for its excellent activity and solubility compared to other counterions, such as NaOH and LiOH [98]. In addition, alkanolamines, such as MEA and methylaminopropylamine (MAPA), can also act as neutralizing agents [99]. When amino acids are combined with MEA, the amino group of the amino acid becomes primarily responsible for CO_2_ absorption. By contrast, the amino group of MEA facilitates the deprotonation of the amino acid, thus improving the absorption capacity, which is an attractive approach for mixed solvent CO_2_ absorption systems [99].

Organic salts from amino acids show CO_2_ absorption properties comparable to alkanolamines, such as MEA, at equivalent concentrations. Furthermore, combinations of amino acids with alkanolamines tend to be more stable than amine solutions alone [100]. On the other hand, salts formed with inorganic bases. such as KOH. exhibit inferior performance in CO_2_ absorption compared to amino acid-amine salts. This highlights the importance of the type of base used in the solution in determining the final absorption capacity. Additionally, external factors, such as temperature and partial pressure of CO_2_, also influence this capacity; as the temperature increases, the solubility of CO_2_ decreases because absorption is an exothermic process [100]. Although mixtures of amino acid salts and alkanolamines improve both absorption and thermal stability, the restrictions imposed by chemical equilibrium limit their practical application in industrial environments where CO_2_ capture is required at high temperatures, such as in industrial chimneys.

#### 3.5.2. CO_2_ Adsorption Through Porous Materials

A crucial aspect of CCS (carbon capture and storage) is using porous materials that facilitate CO_2_ capture [101]. The first mesoporous silica-based material was published in 1990, named M41S [102]. This material served as the raw material for the development of new materials with attractive properties, such as SBA-15 (a mesoporous structure with diameters ranging from 6 to 30 nm, which provides a high specific surface area), SBA-16 (unlike SBA-15, this material features a three-dimensional pore distribution that enhances access to the active sites), FDU-2 (with adjustable pore sizes, allowing for improved capture capacity of various pollutants), MCM-41 (with a distribution combining micropores and mesopores, which enhances the capture capacity for a wide range of contaminants with varying sizes), MCM-50 (with a hexagonal structure similar to MCM-41, its hexagonal arrangement improves the diffusion of pollutants within the material and offers high thermal stability), and KIT-5 (featuring larger pores compared to the previous materials, with pore sizes exceeding 30 nm, which is of interest for increasing diffusion and adsorption capacity) [103]. These materials have been modified for various industrial applications, achieving pore sizes ranging from 0.7 to 70 nm, a surface area of around 1000 m^2^/g, and excellent thermal stability [104]. The synthesis and modification of porous materials has become an attractive approach for treating various industrial waste fluids [105]. Additionally, these porous materials have been modified through functionalization and the grafting of amines into their structure to enhance their reactivity towards CO_2_ and facilitate their scalability for mitigating CO_2_ accumulation in local and mobile sources by transforming it [106,107]. In this regard, various materials with controlled porosity have been developed to facilitate the diffusion of a wide range of fluids, which may exhibit sensitivity and high reactivity towards CO_2_ depending on their structure, enabling them to capture and transform pollutant gases efficiently [108].

An interesting material reported is a mesoporous molecular sieve, known as MCM-41, a silica-based porous support with excellent porosity employed in removing various contaminants, such as heavy metals, from wastewater [109]. However, it exhibited a low capacity for adsorbing and transforming gaseous pollutants, such as CO_2_, due to the lack of reactive groups that can interact with the gas and capture it, limiting its scalability in highly industrialized sectors for carbon capture. Furthermore, the synthesis of these porous materials uses precursors, such as tetraethylorthosilicate (TEOS) and surfactants, to form porous structures, which increases production costs, limiting their industrial scalability, including high production costs, difficulties in quality control, and raises concerns about environmental sustainability due to their poor recyclability [110].

##### CO_2_ Absorption Through Functionalized Porous Materials

**Figure 11:**
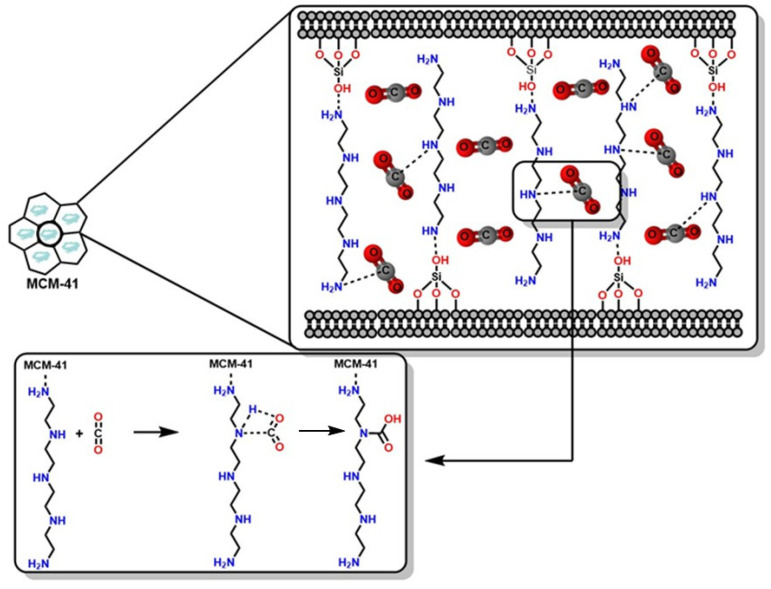
The CO_2_ absorption mechanism using MCM-41 is functionalized with PEI, which expands its CO_2_ capture capacity [115].

The functionalization of materials, such as MCM-41 with 75% Polyethylenimine (PEI), makes this material useful for carbon capture and storage (CCS), demonstrating a maximum CO_2_ absorption of 215 mg of CO_2_/g at room temperature. This result is significant because MCM-41 improves the thermal stability of PEI during heating processes for its regeneration or recyclability; furthermore, it is of interest because it expands the material’s use under high-temperature conditions, such as in industrial chimneys, which represents a significant advantage compared to MCM-50, as mentioned in the previous section [111]. In this regard, Figure 11 shows the functionalization of MCM-41 with tetraethylenepentamine (TEPA) and the CO_2_ absorption mechanism via amino groups present in the porous MCM-41 material. This functionalization increases the number of highly reactive amino groups toward CO_2_ in the material’s pores, generating more selectivity, reactivity, and efficiency in capture and transformation [112]. The capture mechanism involves a nucleophilic attack of the nitrogen atoms in the functionalized material towards the carbon atoms of CO_2_. When the absorbent (porous material) and the absorbate (CO_2_) approach sufficiently, the linear geometry of the CO_2_ molecules is deformed in the presence of the electronic cloud from the nitrogen atoms of the amino groups in the material, inducing a dipole on the linear geometry of CO_2_. Subsequently, a hydrogen atom in the amino group migrates to an oxygen atom in CO_2_, forming an -OH group, resulting in a carbamic acid or carbamate derivative [113]. However, these materials face significant limitations for industrial scalability, such as recyclability. Once these materials reach their absorption saturation, releasing the formed compounds becomes problematic and requires the application of high temperatures, which alters the material’s structure and degrades the compounds formed from CO_2_ absorption [114].

Table 4 reports other attractive absorbent materials for carbon capture, characterized by their moderate porosity. These materials are appealing in rudimentary CO_2_ capture technologies because they exhibit good mechanical properties, are reusable, and prevent the thermal degradation of various amines used for CO_2_ capture and transformation, making them an interesting material for industrial scalability [116]. The structure, regeneration, and CO_2_ capture mechanisms of these technologies are also critical. For example, the functionalization of silica supports TEPA when polyethylene glycol (PEG), a polymer frequently used as a solvent in various formulations, is employed as a solvent, increases the solubility of TEPA and diffusion within the material, as well as the solubility of CO_2_. However, it decreases the CO_2_ absorption capacity due to the accumulation of PEG around the -NH_2_ groups of TEPA, which limits the reaction with CO_2_ [117]. These are key aspects to consider when applying these technologies in industrial sectors.

Depending on the scalability of these technologies, one crucial aspect is the synthesis method. For industrial applications, one-pot synthesis methods are attractive [118]. This synthesis approach minimizes operational time and costs, enabling implementation in industrial sectors with a favorable efficiency/cost ratio at reduced times [119]. Another innovative approach involves multiple functionalization methods within the material’s pores. Specifically, these mesoporous supports can be functionalized by grafting CO_2_-sensitive short-chain amino acids (such as cysteine) into their structure and impregnation with an amine (e.g., TEPA). This method has shown up to a 20% improvement in CO_2_ absorption capacity compared to the material without the amino acid graft, i.e., only impregnated with TEPA [120]. Additionally, reducing the surface area by forming millimeter-sized spheres with hierarchical porosity is also an attractive approach for CO_2_ capture, as it increases the surface area and expands the channels, facilitating diffusion, as seen with the SSF-PEI60 material. However, this material presents severe limitations regarding amine concentration, as it has been shown that, at 70% PEI concentration, it performs less effectively in CO_2_ capture compared to 50% PEI concentration, where it records absorptions of up to 188.3 mg of CO_2_/g of absorbent. This decrease is due to the obstruction of the material’s channels caused by the high viscosity of PEI at high concentrations, which limits the absorption capacity of PEI within the material [121]. In this regard, it is important to highlight these limitations to optimize these materials’ CO_2_ absorption and capture capacity and ensure their industrial scalability.

On the other hand, the PEI–MSP-0.32 material is attractive in CO_2_ capture in industrial sectors due to its easy functionalization with amines, thanks to its porous structure and active groups that can form bonds with amines, enhancing carbon capture. It exhibits good mechanical properties and recycling potential, making it an appealing material for industry [112,122]. In this context, it is clear that these materials are attractive for CO_2_ capture but have some limitations, such as the low amine concentrations required to achieve optimal diffusion within the material and efficient gas diffusion (which reduces CO_2_ absorption capacity), as well as the type of solvents used to dilute the amines and moderate costs. This is important because using moderate amine concentrations (<70%) offers a good economic fit, which is attractive for industrial applications but decreases the CO_2_ absorption capacity [123]. In this regard, the importance of amino groups in the structures of materials to ensure their efficiency in CO_2_ capture is evident, as well as the use of moderate concentrations of organic solvents in the impregnation method due to their viscosity, which limits their diffusion within the material and the solubility of CO_2_ in the solution.

**Table 4:** Porous supports with CO_2_ absorption potential and easy functionalization for industrial scalability.

Porous Material	Absorption Capacity (mg/g)	Synthesis	Mechanical Strength (MPa)	Ref.
PEI–MSP-0.32	144 ± 2.0	One pot	0.50	[122]
PEI–MCM-41	76.0	One pot	5.50	[124]
PEI–SBA	118 ± 4.0	One pot	0.60	[122]
PD–PEI	53.7	3D printing	0.15	[125]
PEI–MCM-P	103.8	One pot	0.46	[126]
PD–TEPA	98.1	3D printing	0.33	[125]
5A–R4	60	3D printing	0.35	[127]
13X–R4	61	3D printing	0.69	[127]
70T–MM-550	151.1 ± 2.8	Sol gel	4.66	[112]

##### Adsorption of CO_2_ Through Polymeric Materials

Polymeric materials offer distinct advantages compared to the materials discussed in the previous section. For instance, polymeric materials can be synthesized to incorporate -NH_2_, -NH-R_1_, and R_1_-N-R_2_ groups into their structure; this advantage eliminates the need for further functionalization applied to the porous materials mentioned earlier [128]. Although these materials lack the periodicity of pores due to their three-dimensional network structure, they possess a large surface area, which is attractive for carbon capture technologies using amine impregnation [129]. In this regard, various polycondensation reactions have been reported, such as radical polymerization [130], Schiff base condensations [131], substitution reactions [132], Friedel–Crafts reactions [133], and diazo coupling reactions [134], among others.

These polymeric material synthesis methods not only enable the production of materials with CO_2_ capture potential, which can be used in the fight against anthropogenic CO_2_ accumulation in the environment (as they function as molecular scaffolds for supporting amines), but they allow for their valorization, thereby highlighting their relevance in sustainable strategies for mitigating environmental impact through industrial scalability [135]. The presence of amines in these polymers has been shown to improve their adsorption capacity significantly, optimizing their performance in industrial applications [136]. Moreover, the polymers used in these materials can be biodegradable, which, being non-toxic and non-polluting, presents an attractive eco-friendly alternative for industrial scalability [137].

**Figure 12:**
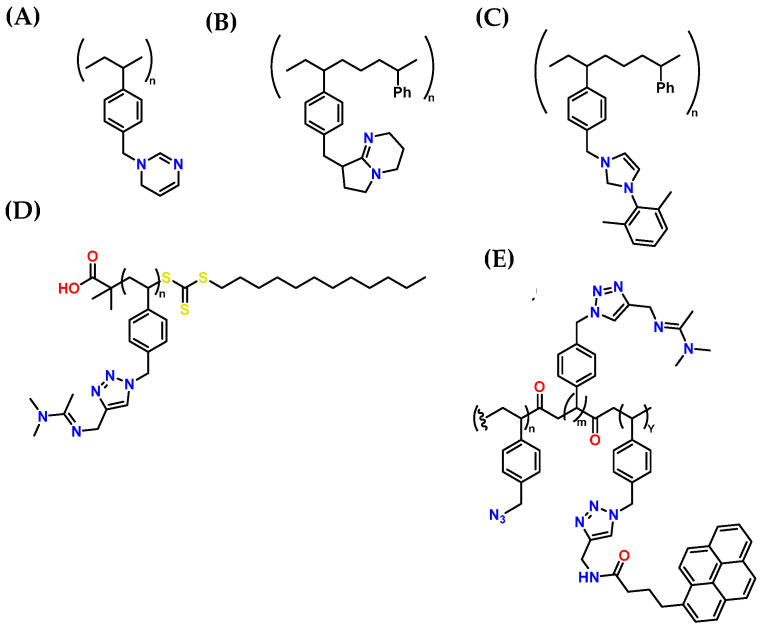
Molecular structures of potential polymers for CO_2_ adsorption: (**A**) N-Methyltetrahydropyrimidine. (**B**) 1,5-diazabicyclo [4.3.0]non-5-ene. (**C**) N-heterocyclic carbenes. (**D**) N,N-dimethylacetamidines. (**E**) Amidine-based copolymer [38].

One polymer of interest for CO_2_ capture and storage is N-methyl tetrahydropyrimidine (MTHP), which contains amidine groups capable of reacting with CO_2_ reversibly in solution and solid state. The structure of MTHP includes an amidine unit incorporated into a polymeric matrix, enhancing its CO_2_ adsorption capacity [108]. Figure 12A illustrates a polystyrene derivative containing an amidine fraction, known as poly(THPSt). This structural design improves CO_2_ adsorption efficiency and highlights the crucial role of amines and their derivatives in developing innovative polymeric materials for climate change mitigation applications [108].

Due to their high porosity, the diffusion of gases and liquids is facilitated (which is helpful for gas capture through amine impregnation methods, i.e., CO_2_ capture with low-concentration amine solutions). They allow release at lower temperatures than carbamates formed by CO_2_ absorption using primary or secondary amines, making them particularly relevant for recyclability [138]. To further enhance CO_2_ adsorption capacity, the functional comonomer N-vinylacetamide (NVA) has been incorporated into the polymer matrix (Figure 12B), optimizing CO_2_ adsorption. In this context, the copolymer poly(THPSt50-co-NVA50) (Figure 12C) has shown a significant improvement in CO_2_ adsorption compared to pure poly(THPSt) [139]. This improvement is attributed to the more excellent CO_2_ permeability of poly(NVA). Therefore, this type of polymer has fantastic potential for CO_2_ storage and transformation, contributing to removing CO_2_ from industrial waste gases. An innovative polymer based on N-heterocyclic carbene (NHC) fractions has been developed, showing notable potential for CO_2_ adsorption (Figure 12D). This material is distinguished by its high reactivity toward CO_2_, attributed to its ability to form larger pores. This characteristic facilitates gas diffusion, allowing for more efficient capture even at low CO_2_ concentrations [38].

Furthermore, polymers based on amidine with moderate hydrophobicity are synthesized using RAFT polymerization and “click” chemistry (Figure 12D). However, this synthesis approach is unfavorable for ensuring industrial scalability due to the controlled conditions and high costs involved [140]. Additionally, a critical feature of this polymer is that it is insoluble in water but soluble in solvents, such as N,N-dimethylformamide, trichloromethane, dichloromethane, and dimethyl sulfoxide, which could enhance CO_2_ adsorption in non-aqueous solvents through amine impregnation methods. However, the costs would be very high for industrial applications [141]. Likewise, CO_2_-sensitive copolymers modified with pyrene (Figure 12E) have significantly improved the carbon diffusion within their structure [142]. Several authors have reported the synthesis of these copolymers with varying amidine group ratios, which improves the basicity of the material toward acidic gases like CO_2_ [143].

The dispersibility of these copolymers shows a significant improvement in solvent dispersions, such as dichloromethane and N,N-dimethylformamide. This result is attractive for CO_2_ capture using the amine impregnation method due to their moderate polarity and favorable physical interactions with the solvents, optimizing diffusion within the polymer [144]. Furthermore, the transition between hydrophobic and hydrophilic states in mixtures of dichloromethane/water (1:1, *v*/*v*) when exposed to CO_2_ causes an observable physical change manifested through fluorescence, indicating effective CO_2_ absorption by the system. These findings highlight the potential of copolymers for applications in technologies that require efficient manipulation and dispersion of carbon in liquid media [143]. However, a common feature of these materials is their synthetic origin, which presents challenges for industrial scalability because their mass production can generate accumulation and contamination. In addition, there are no exhaustive studies on their recyclability, mechanical behavior, and thermal stability; the lack of these studies is risky for scaling these materials.

##### Adsorption of CO_2_ Through Macrocyclic Polymeric Materials

The synthesis of polymeric materials for industrial scaling has emerged as an exciting area in chemistry as a multidisciplinary approach, with particular emphasis on polycondensation reactions that allow the formation of complex structures, including interconnected macrocyclic rings through aromatic substitutions [145]. For example, the polycondensation reaction of triazine containing trialdehyde and pyrrole results in a porous polymer with promising applications in scaffold design; the stability of this polymeric network is enhanced by electrophilic aromatic substitution reactions under acidic conditions, where the Fe (III) ion plays a crucial role, improving the CO_2_ adsorption properties of the material. These macroporous materials are attractive for the scalability of CO_2_ adsorption technologies using chemical solvents because they allow greater diffusion of amines; that is, they enable the use of more concentrated amines (>70%), thus enhancing adsorption capacity and improving CO_2_ capture [146].

**Figure 13:**
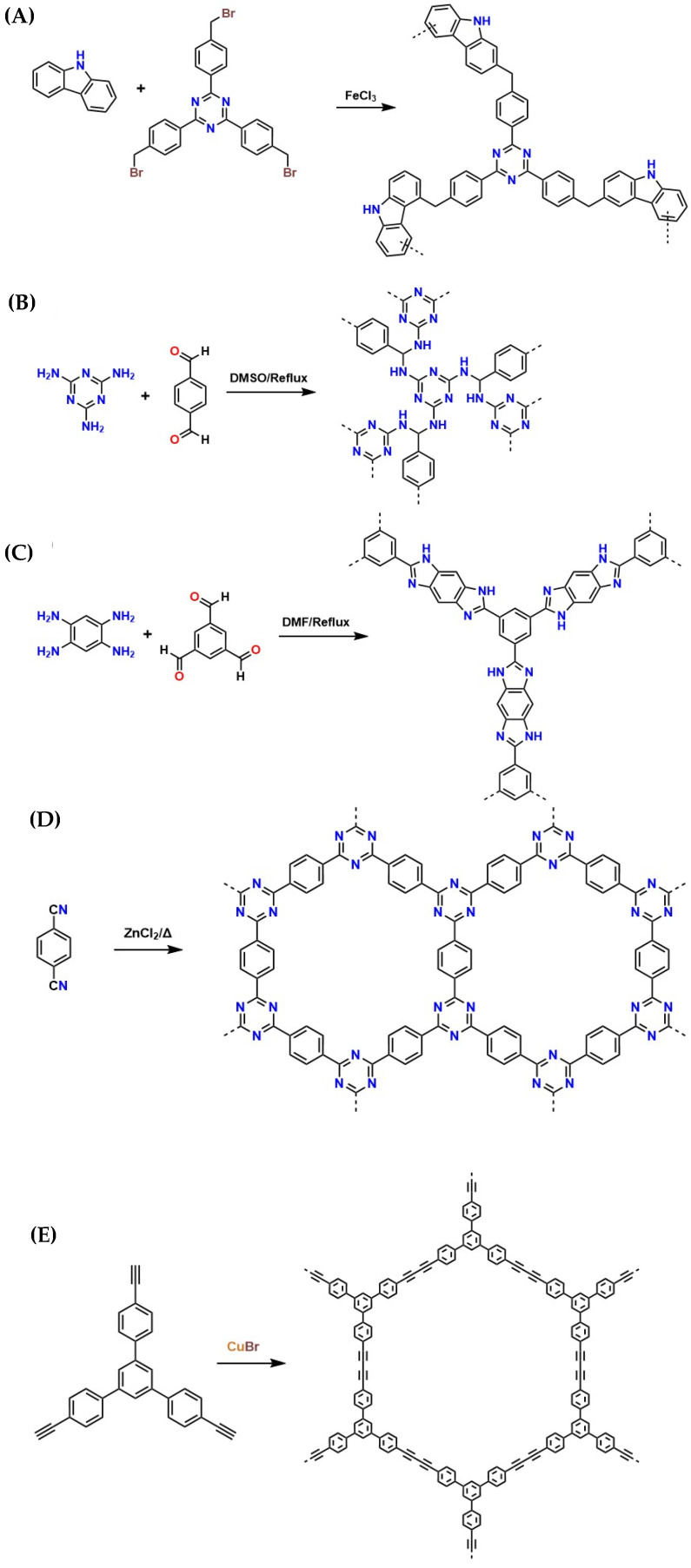

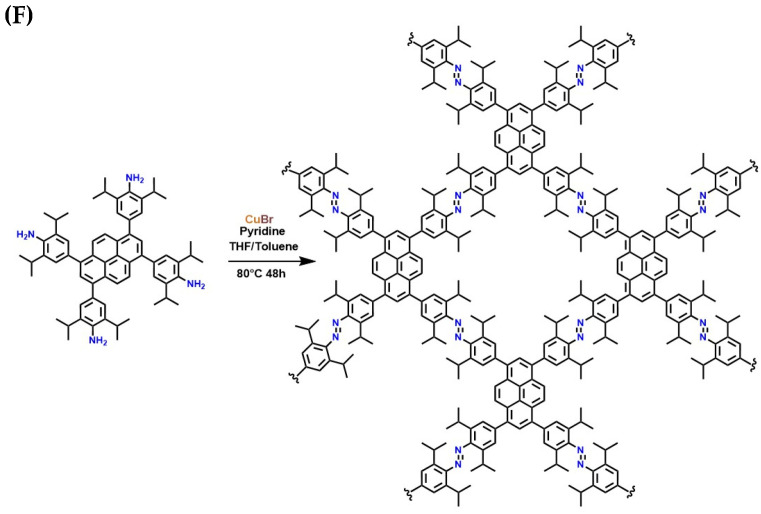
Synthesis of porous organic polymers with good diffusion capacity and high mechanical strength. (**A**–**C**) polymers linked (functionalized) with benzimidazole. (**D**–**E**) 1,3,5-tris-(4-ethynylphenyl)-benzene. (**F**) Py-azo-COP material [151].

Figure 13 summarizes some of the key syntheses used in producing organic polymers. Additionally, hypercrosslinked polymers (HCPs) can be synthesized through Friedel–Crafts reactions between a halide and various activated aromatic compounds. For instance, the synthesis of an HCP (Figure 13A), where 2,4,6-tris [4-(bromomethyl)phenyl]-1,3,5-triazine undergoes a Friedel–Crafts alkylation with carbazole in the presence of AlCl_3_ to produce the corresponding porous crosslinked polymer.

Figure 13B shows a typical example of a polymeric material synthesis through Schiff base condensation for the reaction between melamine and terephthalaldehyde, while symmetrical amines and aldehydes result in crystalline porous polymers. Asymmetric or branched monomers lead to the formation of amorphous porous polymers [147]. Furthermore, polymer synthesis (Figure 13C) coupled with benzimidazole, where a tetraamine containing multiple ortho-amino groups reacts with an aromatic polyaldehyde containing two or more aldehyde groups [148]. These polymers are of great interest in developing sustainable strategies for CO_2_ adsorption due to their highly porous structures, which, after a lyophilization process, serve as scaffolds to support a large amount of amines potential for CO_2_ adsorption. This result overcomes the limitation of using low-concentration amine solutions to prevent pore clogging due to their viscosity [149,150].

In this context, research based on new potential technologies for the safe capture of carbon and the recyclability of both the porous material and the adsorbed gas has led to the synthesis of covalent triazine frameworks (CTFs), which are another class of porous materials synthesized through the cyclotrimerization of cyanobenzenes in the presence of ZnCl_2_ and anhydrous conditions (Figure 13D). These polymers are interesting materials due to their high liquid retention capacity, making them suitable for supporting amine solutions [151]. Additionally, aromatic compounds containing multiple cyano groups (either symmetric or asymmetric) can undergo cyclotrimerization in each of the cyano molecules, thus leading to various CTFs. Because there are many N-sites in the CTFs, they often show good CO_2_ uptake and diffusion capacities. They are frequently used as supports to stabilize ultra-small metallic nanoparticles on the surface of these porous polymers [151].

Another important class of macroporous materials is π-conjugated systems, which can be synthesized through a wide range of homocoupling and cross-coupling reactions between aromatic halides (containing multiple halogenated sites, mainly bromides due to their higher reactivity and convenient preparation) with boronic acids, alkenes, alkynes, among others. Figure 13E shows a typical example of the CuBr-catalyzed homocoupling of a terminal aromatic alkyne, 1,3,5-tris-(4-ethylphenyl)-benzene [151]. Furthermore, it exhibits an excellent gas storage capacity due to extensive π-conjugation, demonstrating that these materials are promising for the design of new materials for CCUS [152]. On the other hand, extended aromatic substitution in two or more positions on the aromatic ring can lead to the formation of the porous network, as shown in Figure 13F for the synthesis of macroporous polymers through acid-catalyzed polycondensation reactions between pyrrole and terephthalaldehyde [146].

In addition to all the advantages that these materials present, they are not exempt from conditions that are prohibitive for their industrial scalability. For example, the synthesis of these materials involves high costs, and there are no exhaustive studies on their thermal behavior, mechanical stability and degradability. These conditions can be prohibitive and limit their application in sectors with high CO_2_ emission rates. Future research should expand the applications of these materials and develop prototypes that exhibit good performance under these conditions that are prohibitive for the scalability of these promising materials. CO_2_ adsorption through biodegradable hydrogels.

The structure of hydrogels could exhibit a good capacity for CO_2_ adsorption. In other words, these polymeric materials can also be synthesized to contain the -NH_2_ group in their structure to enhance their basicity towards CO_2_ [153]. Unlike the polymeric materials discussed in the previous section, these materials are crosslinked, which involves the formation of covalent bonds (for example, through the use of glutaraldehyde) [154], or physical crosslinking, which consists of electrostatic interactions (such as the use of polyphosphate) [155], forming three-dimensional structures capable of retaining large amounts of liquids [156].

**Figure 14:**
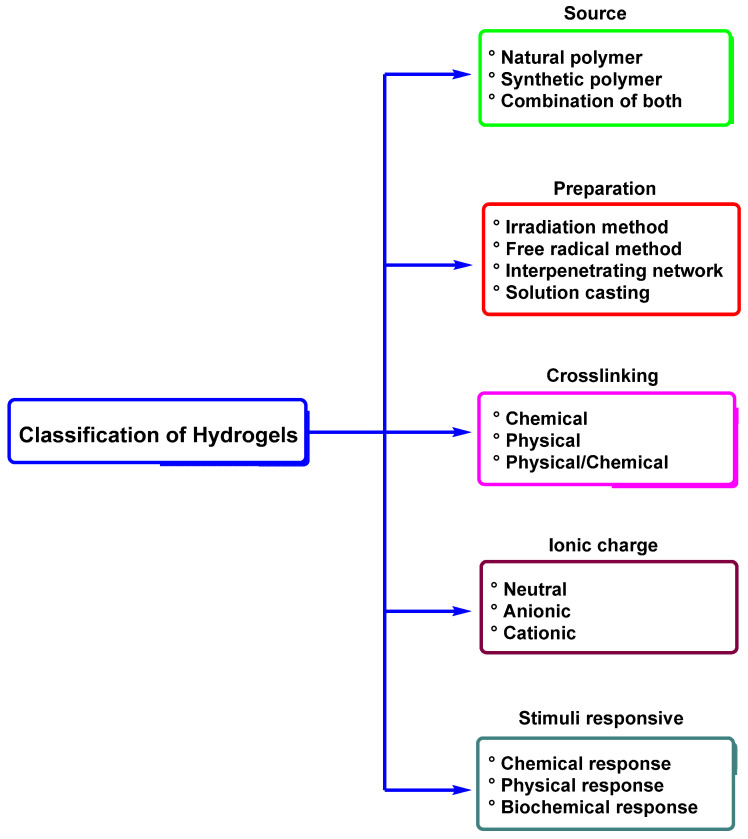
Types of hydrogels and their classification according to the source of origin [157].

These materials are classified based on various factors, as shown in Figure 14. This classification is based on the nature of the polymers that constitute the hydrogel, that is, the source of the polymers (natural or synthetic), the crosslinking method (physical, chemical, or both), their ionic charge (cationic, anionic, and neutral), their response to external stimuli, and the combinations of natural and synthetic polymers [157].

There are fundamental differences between hydrogels and the polymeric materials discussed in the previous section. For example, although there are similarities in the synthesis of polymeric porous materials, hydrogels (especially biodegradable ones) offer more modification options. Additionally, their natural character facilitates recyclability and prevents accumulation [158]. Furthermore, it is possible to control their porosity by varying the crosslinking agents’ concentration. This is interesting because, through the concentrations of the crosslinking agent, it is possible to control the flexibility, mechanical properties, and pore size. Moreover, by adjusting the pH, the charge of polyelectrolyte hydrogels can be controlled [157]. These results are promising for synthesizing materials with moderate porosity and flexibility for CO_2_ capture through impregnation with amino acid salt solutions. For example, glycine, alanine, and sarcosine salts with KOH have increased CO_2_ capture efficiency by up to 30% [159]. Similarly, mixtures of amino acid salts consisting of glycine, alanine, proline, and lysine have demonstrated a 43% increase in CO_2_ capture [160].

**Figure 15:**
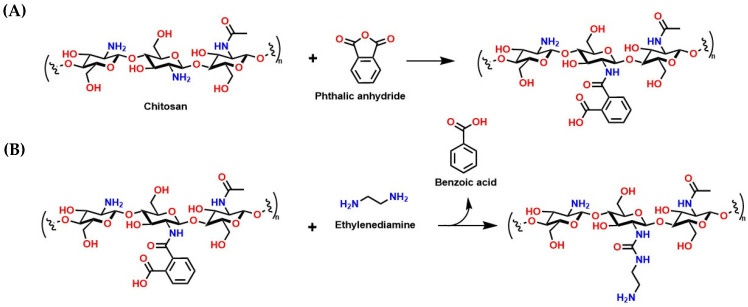
Chitosan functionalization: (**A**) Chitosan functionalization using phthalic anhydride; (**B**) Chitosan derivative functionalized with ethylenediamine, which improves the basicity of the material and increases the -NH and -NH_2_ groups, improving the molecular recognition between CO_2_ and the hydrogel [162].

These crosslinked materials, which can absorb large amounts of liquids and gases, are beneficial due to their high surface area and the ease with which their chemical properties can be modified, making them attractive for incorporating amines into their structure [161]. For instance, a recent study reported the functionalization of a chitosan hydrogel (crosslinked with glutaraldehyde) with phthalic anhydride (Figure 15A) and ethylenediamine (Figure 15B). This functionalization could allow for an increase in the material’s pore size and the number of -NH_2_ groups, which could enhance the CO_2_ adsorption capacity [162]. In this regard, some of the most common synthesis methods include solution polymerization and free-radical polymerization, which produce hydrogels with specific characteristics, such as porosity and elasticity, adapted to environmental conditions [163]. Moreover, these one-pot synthesis methods are attractive for industrial scalability.

However, despite the advantages offered by all the materials discussed so far, the lifespan of these materials in CO_2_ adsorption is short, which presents a significant limitation and concern. One of the main limitations is their long-term stability. Hydrogels have advantages over other materials because they can degrade without generating accumulation or pollution, which is beneficial in industrial applications [164]. The production cost and the complexity of chemical modifications to optimize their performance must also be considered. On the other hand, although hydrogels offer a high initial adsorption capacity, their regeneration after the capture process can be inefficient, requiring additional energy that could counteract or affect the molecular integrity of the material, thus impacting its lifespan and necessitating its disposal. Without proper waste management, this could lead to accumulation (if the material is not biodegradable) and result in a negative environmental impact [165].

**Figure 16:**
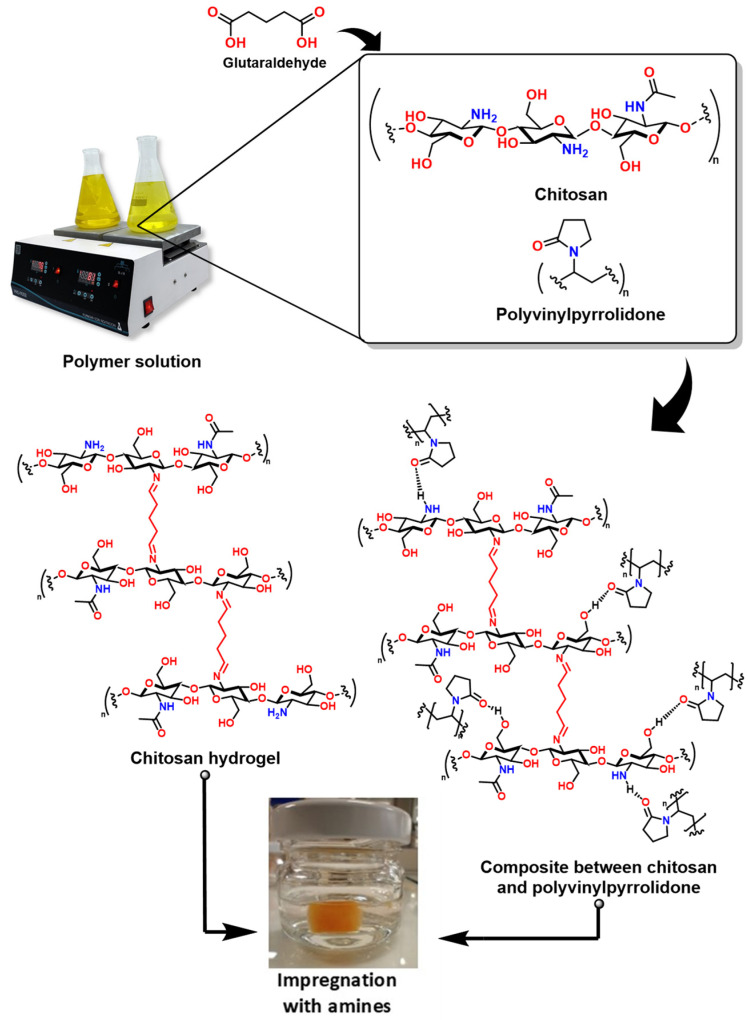
Synthesis scheme of biodegradable hydrogels as promising materials for CO_2_ capture [167].

Moreover, the CO_2_ adsorption ability of these crosslinked materials is limited. For example, our recent theoretical study on molecular recognition between CO_2_ and biodegradable polymers, such as polyvinylpyrrolidone (PVP), 2-hydroxyethyl acrylamide (HEAC), polyethylene glycol (PE), and chitosan, revealed that these materials adsorb CO_2_, with binding energies ranging between −4.5 and −6.5 kcal/mol, indicating physisorption, and a release of the gas at temperatures of 160 °C [113]. This suggests that the material alone (the xerogel) is not viable as a scalable CO_2_ capture technology. Fortunately, the capture capacity of these materials can be enhanced by impregnation with concentrated amines and the incorporation of amines into the xerogel structure, as shown in Figure 16, facilitating industrial scalability.

The amine impregnation method is relevant because it allows for higher CO_2_ solubility and subsequent reaction with amines, which could lead to the formation of a carbamic acid [166]. These hydrogels are effective for applications in the controlled capture and release of compounds and offer significant advantages in environmental sustainability [163].

##### CO_2_ Adsorption Through MOF’s

Metal–organic frameworks (MOFs) stand out as highly efficient materials for the adsorption of CO_2_ due to their extensive surface area, surpassing other porous materials, such as activated carbon and zeolites [168]. The ability of these compounds to capture CO_2_ is directly related to their surface area, which maximizes interaction with gas molecules. In recent decades, advancements in research have enabled the development of MOFs with innovative structures and porosities, such as square channels (Zn_2_(1,4-benzenedicarboxylate)) and open metal sites (Cu_2_(3,3′,5,5′-biphenyltetracarboxylic acid) and Cu_3_(benzenetricarboxylate)_2_) [169], optimizing their performance in CO_2_ adsorption. Additionally, some MOFs exhibit unique characteristics, such as hexagonally packed cylindrical channels (Zn_2_(2,5-dioxo-1,4-benzenedicarboxylate)) and pores functionalized with amino groups (Zn_4_O(2-amino terephthalate)_3_) [170]. Ultra-high porosity frameworks, such as Zn_4_O(1,4-benzenedicarboxylate)_3_ and Zn_4_O(4,4′,4′′-benzenetriyl-tribenzoate)_3_ [170], are particularly notable for their ability to adsorb up to 60% by weight of CO_2_ at 35 bar pressure.

**Figure 17:**
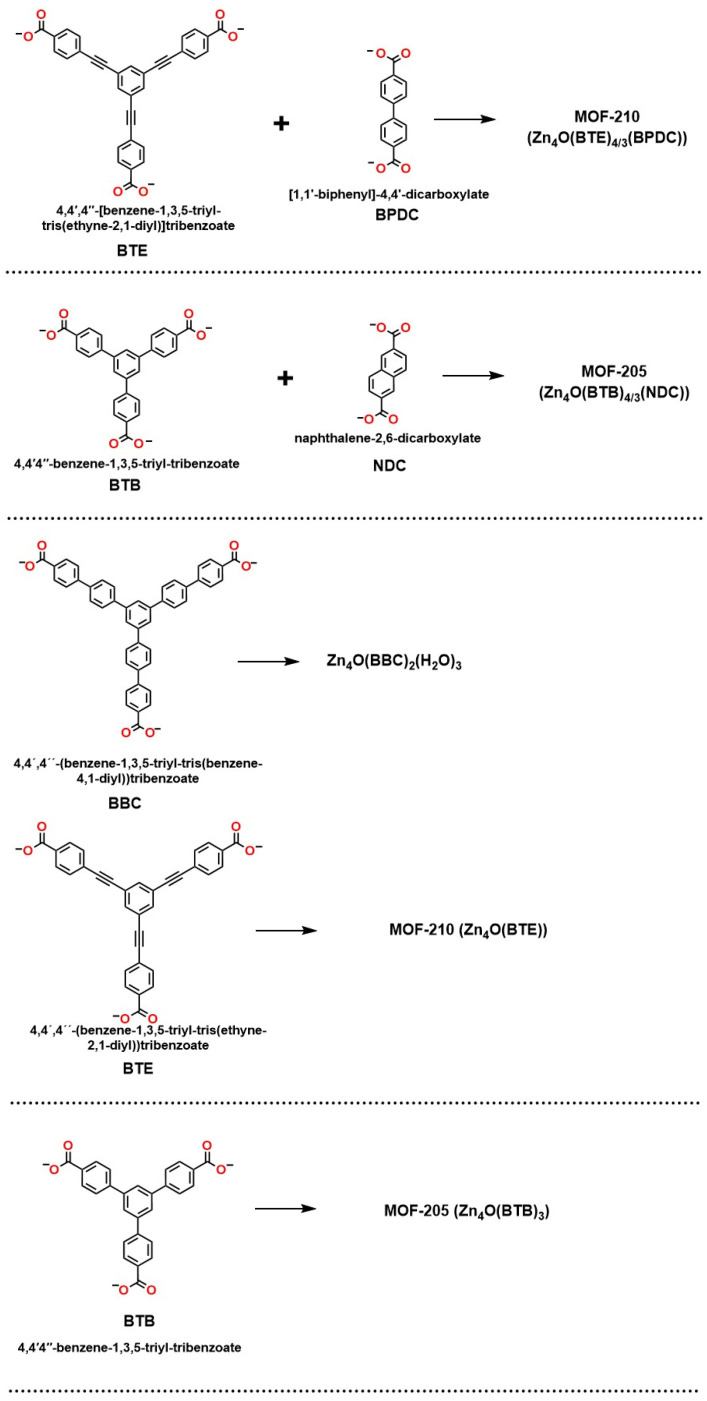
Synthesis of high-porosity MOFs using repeating Zn_4_O(CO_2_)_6_ units as the structural unit [168].

Figure 17 summarizes some ultra-porous MOFs built with Zn_4_O(CO_2_)_6_ units, which is a fundamental compound in coordination chemistry due to its ability to act as a structural unit in MOFs. Its geometry allows efficient interaction with ligands, favoring the formation of complex networks. In addition, it stands out for its high CO_2_ adsorption capacity, which positions it as a key material in carbon capture technologies. These properties make it relevant both in environmental applications and in the development of advanced materials.

These materials have proven highly effective in gas separation processes, essential for carbon capture and storage (CCS) [71]. These processes encompass various applications, such as the separation of CO_2_/N_2_ in post-combustion capture, the separation of CO_2_/H_2_ in pre-combustion capture, as well as air separation (O_2_/N_2_) and CO_2_/CH_4_ in natural gas or synthesis gas enhancement [71]. Adsorption capacity and selectivity are crucial in these separations, as they directly influence process efficiency. Furthermore, understanding the CO_2_ adsorption sites within MOFs and the binding mechanisms is vital for designing new materials that optimize these properties. For example, it has been observed that MOFs with unsaturated metal centers, such as Mg-(2,5-dioxo-1,4-benzenedicarboxylate) [170] and Cu_3_(benzenetricarboxylate)_2_ [169], exhibit significant electrostatic interactions with CO_2_, making them key points for its adsorption.

Studies have revealed that, in the case of Mg-(2,5-dioxo-1,4-benzenedicarboxylate), all CO_2_ molecules bind to the open Mg^2+^ site, while in Cu_3_(benzenetricarboxylate)_2_, most of the CO_2_ adheres to the open Cu^2+^ sites at low concentrations [171]. However, a high charge is required to allow large amounts of CO_2_ to enter the small windows of the structure. In adsorption sites not associated with unsaturated metal centers, van der Waals interactions predominantly maintain the adsorbed CO_2_. These findings not only enhance the effectiveness of MOFs in gas separation but open new opportunities for their use in advanced CCS technologies, significantly contributing to the fight against climate change [171].

#### 3.5.3. Emerging Technologies for CO_2_ Capture

CCUS and CCU are technologies developed to combat the accumulation of CO_2_ from local sources, such as the energy, industrial, and transportation sectors, without disrupting the normal production processes in these sectors, which are crucial for the economic stability of many regions and countries. The difference between CCUS and CCU lies in the final destination of the captured CO_2_. In this regard, CCU stores the captured CO_2_ in geological formations, raising concerns about its long-term effects and the accumulation associated with this technique. CCUS is more widely accepted and presents a more attractive circular economic approach for industrial applications. This approach transforms the captured CO_2_ into value-added products, such as carbamates [172,173,174]. These technologies have positioned themselves as key solutions in the fight against climate change, designed to mitigate CO_2_ emissions across various industrial sectors, as summarized in Figure 16 [175]. Therefore, these technologies offer an appealing approach to mitigating CO_2_ accumulation and global warming [176]. In industrialized sectors, capture can be achieved through post-combustion, pre-combustion, and oxy-fuel combustion, each adapted to different contexts [177].

**Figure 18:**
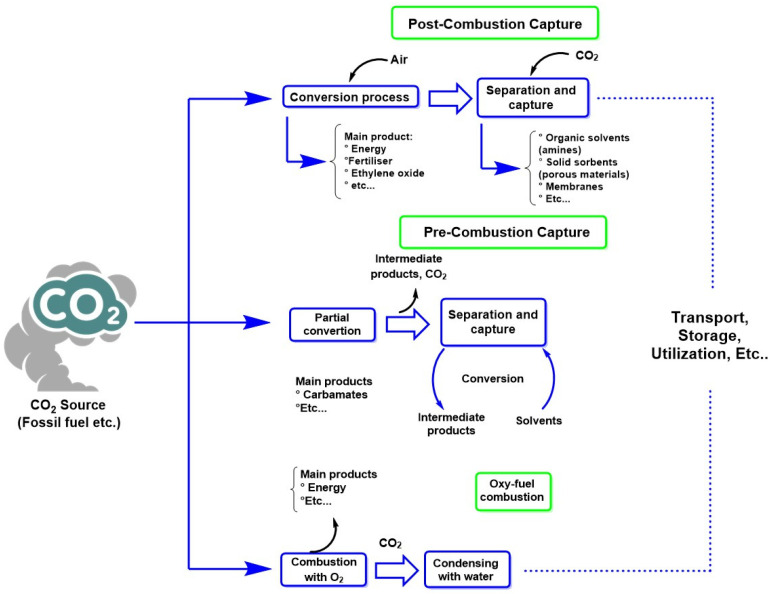
CO_2_ capture technologies in industrial sectors [177].

Figure 18 summarizes the capture methods: post-combustion capture is one of the industrial sector’s most advanced and widely used technologies to mitigate greenhouse gas emissions [178]. This method, implemented for several decades, relies on separating CO_2_ from other combustion gases using chemical substances, with amines being the most commonly used solvents due to their effectiveness [179]. The main advantage of post-combustion capture is its adaptability, as it allows various emission sources, such as power plants and industrial facilities, to be equipped with separation systems. Despite its ability to remove up to 85% of the CO_2_ in the gases, this process incurs high costs and significant energy consumption, posing challenges for large-scale implementation [180].

Pre-combustion capture is also an innovative technique for reducing CO_2_ emissions by breaking down volatile hydrocarbons, such as natural gas, into H_2_ and CO_2_ before combustion, allowing for selective CO_2_ capture [181]. Based on coal gasification or natural gas reforming, this process is essential in various industrial applications, especially in ammonia production and energy generation using hydrogen-rich gases [182]. The output of H_2_ is of increasing interest because H_2_ is an efficient fuel; its combustion does not produce toxic gases, and it holds promise for decarbonizing important industrial sectors [183].

In addition, oxy-fuel combustion has been reported as one of the most prominent methods for CO_2_ capture in the industrial sector. It is characterized by the use of oxygen, typically extracted from the air, for the combustion of hydrocarbons of economic or energy interest [184]. This process generates a residual gas mainly composed of water vapor and CO_2_, which can be separated through cooling, allowing the condensation of the water vapor and subsequent extraction of the CO_2_. Oxy-fuel combustion produces high temperatures, requiring the adaptation of technologies to capture residual combustion gases, such as CO_2_ [184]. However, this method can offer superior energy efficiency compared to conventional plants that use air; separating and compressing air results in high energy consumption, leading to a 12% reduction in overall efficiency [185]. Thus, power plants employing oxy-fuel combustion and CO_2_ compression achieve 43% and 48% efficiencies. One of the main advantages of oxy-fuel combustion is its ability to capture nearly all CO_2_ generated without releasing other pollutants, making it an attractive option for industrial sustainability.

**Figure 19:**
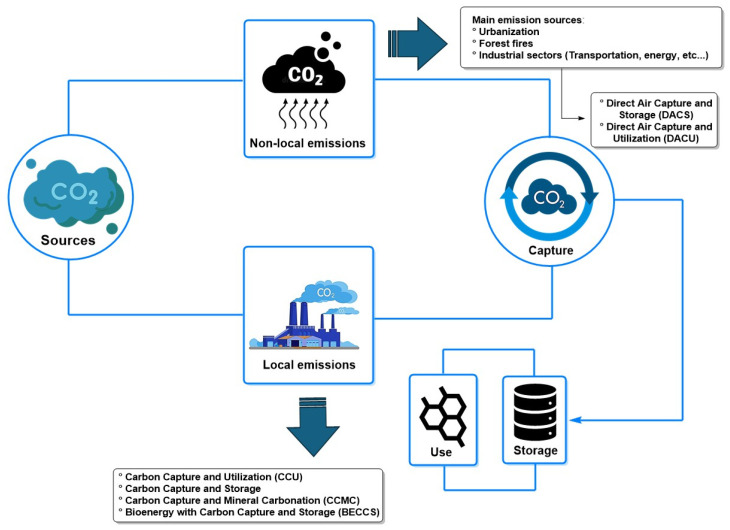
Standard scalable technologies for CO_2_ capture, storage, and transformation [175].

Figure 19 summarizes the applications of carbon capture technologies in the industrial sector; however, these technologies face significant challenges in general, such as high capture and transportation costs and their potential to reduce emissions [186]. As investments increase and new technologies are developed, CCUS is positioned as a key tool for decarbonizing the industrial sector, especially in fossil fuel-dependent countries, offering a pathway to a more sustainable future.

Bioenergy with carbon capture and storage (BECCS) has emerged as an attractive alternative that integrates energy generation from biomass with the simultaneous capture and storage of CO_2_ [187]. According to the Intergovernmental Panel on Climate Change (IPCC) report, this technology has been highlighted as one of the most effective strategies for mitigating anthropogenic CO_2_ emissions from local sources, with a reduction potential ranging from 0.4 to 11.3 gigatons of CO_2_ per year (from 2020 to 2050) [188]. In addition, another competitive alternative, carbon capture and utilization (CCU), focuses on reusing captured CO_2_ as a raw material in industrial processes [189]. This technology helps reduce dependence on fossil fuels and allows the production of valuable new products. Both technologies represent significant advancements in the fight against climate change, offering solutions focusing on emission reduction and transforming CO_2_ into useful resources, thus contributing to a circular and sustainable economy [190].

Although carbon capture technologies are promising for mitigating CO_2_ emissions, their application is mainly limited to local emission sources. This presents a challenge, as CO_2_ generated by non-local sources, such as agricultural waste, the residential sector, municipal waste, and especially transportation, is nearly impossible to capture [191]. Direct air capture (DAC) has been developed to address this limitation, allowing the extraction of CO_2_ that has already been emitted directly into the atmosphere. This technology is divided into two approaches: liquid DAC, which uses solvents to absorb CO_2_, and solid DAC, which employs solid adsorbents [192]. Currently, 19 operational DAC facilities worldwide can capture over 0.01 megatons of CO_2_ per year [193].

The main aspects of DAC include its ability to operate independently of emission source locations, making it a versatile solution for reducing CO_2_ concentrations in the atmosphere. However, its implementation faces significant challenges, such as high costs and energy needs [194]. Furthermore, although DAC can complement traditional carbon capture and storage (CCS) technologies, its price per ton of CO_2_ removed is considerably higher. As research advances and new facilities are developed, DAC could play a crucial role in the global strategy to combat climate change and achieve long-term sustainability goals. Table 5 presents solid adsorbents that show notable potential for industrial scalability in CO_2_ capture and valorization. This approach is fundamental for advancing decarbonization, a key goal in the fight against climate change, and for meeting the Sustainable Development Goals (SDGs), particularly SDG 13 (www.globalgoals.org/goals/13-climate-action/ (accessed on 12 December 2024)), which focuses on climate action.

Table 5 summarizes the porous materials used in scalable carbon capture technologies, a key topic in the fight against climate change [195]. However, adverse environmental conditions may affect the long-term stability of these infusions, which could limit their effectiveness in industrial applications.

**Table 5:** Scalable technologies for CO_2_ adsorption using potentially scalable solid adsorbents.

Type of Technology	Material	Type of Amine/Polymer	Percentage of Amines/Polymer	Adsorption (mg-CO_2_/g-Material)	Advantages/Opportunities	Disadvantages	Ref.
Molecular sieve	MCM-41–PEI	PEI ^1^	75	215	This mesoporous molecular sieve exhibits a synergistic effect on CO_2_ adsorption with PEI at 75 °C. This material increases the porosity and the diffusion of the gas through it. In addition, it improves its mechanical properties, which allows its recyclability.	Industrial scalability faces significant challenges, including high production costs due to complex synthesis processes and raw material prices.	[25]
Molecular sieve	PEI–MCM-41	DEA ^2^/Silica	25	2.41	PEI–MCM-41 exhibits a high pore volume, allowing for a higher loading level of DEA compared to zeolite 13X. The CO_2_ capacity and adsorption rate of the DEA-impregnated PEI–MCM-41 reached maximum values at loading levels slightly above pore saturation.	The main disadvantages of these materials are high operating costs and incomplete desorption, which restrict their adsorption capacity. Although they have good humidity tolerance, their performance can be affected by variations in the composition of the gas stream.	[196]
Mesoporous silica	SBA-15 (SBA(P))	TEPA ^3^	40	173	This material is adequate for CO_2_ capture and has several advantages. First, it saves energy and time since the material must not be removed. The second advantage is that this material retains a slightly higher capacity for CO_2_ adsorption than the unmodified SBA-15 sample. Third, the existence of P123 in SBA-15 increases the reactivity against CO_2_ due to the presence of TEPA	Although it has a moderate adsorption capacity and good thermal stability, its main disadvantages are the material’s recyclability and high costs, which make it unsuitable for industrial scalability.	[197]
Molecular sieve	SBA-15 (SP)	TEPA/DEA/Sílice	30	144	Including amino groups in TEPA significantly improves the adsorption of CO_2_ on this amine-modified material. The presence of these groups is crucial since their absence would allow two amino groups to react with a single CO_2_ molecule, generating carbamate-type zwitterions. By contrast, adding hydroxyl groups using DEA prevents the formation of zwitterions by allowing only one amino group to react with each CO_2_ molecule.	Although this material has a moderate and long-lasting adsorption capacity, there are limitations to its recyclability and the high costs of the materials for its synthesis. In addition, its poor thermal behavior limits its application in high-temperature sectors.	[198]
Porous material	TEPAN/E-100AN	^4^ MEA	30	296	This material allows moderate diffusion of the impregnated amines and moderate desorption, which is helpful for its recyclability under adsorption/desorption conditions. Furthermore, the initial adsorption and desorption rates shift earlier, and the ability to break the CO_2_ equilibrium decreases slightly when the temperature increases, indicating good desorption. Third, this material’s cyclic reproducibility are superior to those of the other materials tested, such as zeolite 13X.	One of the main disadvantages is the high synthesis costs, which can limit its industrial scalability. Another limitation is the initial desorption temperature at 75 °C, which could also be prohibitive for its application in many sectors.	[199]
Hyperbranched amino silica (HAS)	SBA-15	-	-	140	The pore characteristics of the original SBA-15 support were physical boundaries that limited the number of amines incorporated into the adsorbent and the mass transfer to those amines. This is interesting for the study and design of polymers with specific pores.	In addition to the high production costs, this material requires functionalization to improve its CO_2_ capture capacity. Functionalization increases costs, which could be prohibitive for its scalability.	[200]

^1^ Polyethyleneimine. ^2^ Diethanolamine. ^3^ Tetraethylenepentamine. ^4^ Monoethanolamine.

##### The Promise of DAC in Harmful CO_2_ Emissions

DAC technologies represent a crucial advancement in mitigating climate change by removing carbon dioxide directly from the atmosphere [194]. One of their main advantages is achieving harmful emissions, which is essential for meeting global climate goals, such as those outlined in the Paris Agreement [201]. Additionally, the flexibility of implementation allows these technologies to be deployed in various environments, particularly in areas with access to renewable energy, maximizing their versatility. Compared to natural solutions like reforestation, DAC plants require significantly less space, making them ideal for urban areas or regions with spatial constraints [192].

Innovative applications are also being developed to reuse captured CO_2_, such as producing sustainable fuels or industrial materials, which could generate additional economic benefits and promote a circular economy. However, the future success of DAC will largely depend on technological advancements that reduce operational costs and optimize the use of clean energy [202].

Despite its potential, DAC technologies face significant challenges that limit their global adoption. The current cost per ton of captured CO_2_ remains high, ranging from two to six times above the ideal economic target of less than $100 per ton. This issue is exacerbated by the high energy consumption of the process, especially in systems that require heat at extreme temperatures. While some variants can operate using renewable sources like geothermal energy or heat pumps, many installations still rely on natural gas, which may diminish net benefits regarding emission reductions [202].

DAC technologies are gaining prominence in the fight against climate change, supported by ambitious policies and significant global investments. In the United States, programs like the 45Q tax credit, enhanced by the Inflation Reduction Act of 2022, offer financial incentives of up to $180 per ton of permanently stored CO_2_ [203]. Additionally, $3.5 billion has been allocated to develop four large DAC centers, including Project Cypress in Louisiana and the South Texas DAC Hub, with a combined capacity of over 2 million metric tonnes of CO_2_ annually. Canada and the European Union also advance this technology through national strategies and regulatory frameworks, such as the EU Carbon Absorption Certification Framework (https://www.iea.org/energy-system/carbon-capture-utilisation-and-storage/direct-air-capture (accessed on 12 December 2024)). These efforts reflect a global commitment to scaling DAC technology, which enables net emissions reductions and the reuse of captured CO_2_ in industrial and energy applications. However, DAC technologies face significant challenges, such as high operational costs and elevated energy consumption. Despite these hurdles, initiatives in the United States, Europe, and Canada represent promising steps toward this technology’s scalability and economic viability, which is crucial for achieving global climate goals by 2050 (https://projectcypress.com/ (accessed on 12 December 2024)). This raises questions about its scalability and ability to significantly contribute to global climate goals [201]. However, government and private initiatives drive research and development in this field. In this context, DAC could become a key tool within a comprehensive strategy to combat climate change, provided its current economic and technological barriers are overcome.

#### 3.5.4. Challenges to Overcome

As discussed earlier, emerging technologies for CO_2_ capture face significant challenges that must be overcome for their effective large-scale implementation. One of the main obstacles is the high energy consumption associated with the capture processes, especially in methods like post-combustion carbon capture [175]. For example, techniques that use solvents require a considerable amount of heat to regenerate these materials, increasing operational costs and energy consumption [204]. Furthermore, the scalability of these technologies presents a substantial challenge; massive industrial sources, such as cement plants and power plants, generate large amounts of CO_2_ that need to be managed. This poses logistical and engineering problems that must be resolved to integrate carbon capture into these facilities [177] effectively.

Similarly, the materials used in capture systems are also critical. Absorbents and polymeric materials must be highly efficient and able to withstand multiple cycles of adsorption and desorption without significant degradation [205]. This durability is an active area of research, as efforts are made to improve the effectiveness and longevity of these materials [206]. Another key aspect is the environmental and economic viability of CO_2_ capture technologies. The production of materials like amines or metal-organic frameworks may have ecological impacts that counteract the expected benefits if not properly managed [207]. Additionally, integrating these technologies into existing infrastructure often requires significant upfront investments, which can be a barrier for many industries, particularly without incentives like carbon pricing or government subsidies [189]. For example, global CO_2_ capture projects in industrial sectors such as Future Gen—Jewett, FutureGen—Mattoon, and Great Lakes Energy have required initial investments ranging from $1.5 to $2 million (netl.doe.gov/carbon-management/carbon-storage/worldwide-ccs-database).

Public acceptance also represents a significant challenge, particularly for methods involving long-term CO_2_ storage in underground formations. Concerns about leaks and environmental safety may generate resistance in local communities [208,209]. To overcome these challenges, it is essential to foster continuous innovation in developing new materials, process optimization, and policy frameworks that make CO_2_ capture economically and environmentally viable. Collaboration among governments, industries, and scientific communities will be key to achieving a successful and sustainable large-scale implementation [210].

#### 3.5.5. Final Thoughts on the Future of Carbon Capture Technologies

The future of biodegradable hydrogels and emerging CO_2_ capture and release technologies presents a promising opportunity, although not without significant challenges. These hydrogels, capable of absorbing and releasing CO_2_ in a controlled manner, could represent an essential advancement in carbon capture. Using natural and renewable resources could offer a more sustainable and cost-effective alternative than conventional materials, such as amines or synthetic absorbents [19]. For example, chitosan can be extracted through the partial deacetylation of chitin in an alkaline medium (chitin is the second most abundant biopolymer in nature after cellulose). Chitin is found in the exoskeletons of crustaceans and arthropods, and commercial crustacean waste is expected to increase by 7.28 million tons by 2025 [211]. The utilization of this waste presents an interesting circular economy approach, as chitosan can be obtained and used for the synthesis of biodegradable hydrogels.

One of the main advantages of biodegradable hydrogels is their ability to reduce the long-term environmental impact associated with CO_2_ capture systems. However, collective efforts are still needed to optimize these materials regarding efficiency, durability, and scalability [212]. Further research is essential to improve their CO_2_ absorption properties and stability under operational conditions, particularly in industrial applications requiring large-scale solutions.

On the other hand, emerging CO_2_ capture and release technologies, including porous materials and innovative supports, will significantly benefit from advances in materials science, nanotechnology, and process engineering [213]. To maximize their effectiveness, it is crucial to focus on improving energy efficiency and reducing operational costs while ensuring environmental safety and the scalability of these technologies [214].

## 4. Conclusions

The bibliometric analysis conducted in this study retrieved a total of 903 documents published between 2010 and 2023, highlighting the growing concern over global warming and the impact of environmental disasters, such as storms, torrential rains, and glacier melting. The leading countries involved in carbon capture research are China, the United States, and India, which account for more than 491 of the documents analyzed. This international collaboration is evident in the proximity and size of the nodes in the research networks, indicating an active exchange of knowledge and resources. Despite these advances, there is a notable need to strengthen synergistic efforts between countries to optimize carbon capture and transformation technologies and address the economic challenges that less developed nations face in transitioning to a low-carbon industry.

The literature review reveals strengths and weaknesses inherent in scalable CO_2_ capture technologies. A significant finding in this study shows a considerable increase in materials with the potential for CO_2_ capture and their subsequent transformation into value-added compounds. This approach helps mitigate the growing accumulation of greenhouse gases and promotes a circular economy, which is crucial for achieving long-term sustainable development. Biodegradable polymers offer an alternative to porous materials for carbon capture in energy and industrial sectors, making it an attractive approach to developing scalable technologies. However, to maximize the positive impact of these technologies, governments need to establish funding programs dedicated to the research and development of these innovations. Government support is crucial to accelerate the implementation of sustainable practices and facilitate the widespread adoption of technologies that convert CO_2_ into valuable resources. Such backing would benefit the environment and boost the economy by creating new industrial and commercial opportunities in emerging sectors.

Integrating biodegradable polymer-based technologies for CO_2_ capture and transformation represents a promising path towards a more sustainable future, as does CAD, an attractive technology with great potential for CO_2_ capture, but obstacles such as its economic financing need to be overcome to ensure its global scalability. Combining technological innovation with effective government policies can catalyze a transition towards a more circular and responsible economic model aligned with global sustainability goals.

## Figures and Tables

**Table 1:** Top 10 countries with the highest scientific output related to CO_2_ capture by year (2010–2023) extracted from Scopus and WoS.

Country	Published Documents	Citations
China	323	7404
United States	89	5056
India	79	2407
Australia	42	1564
South Korea	40	707
Malaysia	32	913
Canada	30	1596
United Kingdom	29	929
Iran	23	392
Portugal	5	908

**Table 2:** Top 10 sources of publication on CO_2_ capture.

Journal	Number of Documents	Cite	Journal Impact Factor (Year 2023)
Separation and Purification Technology	48	373	8.1
Chemical Engineering Journal	35	1318	13.3
Industrial & Engineering Chemistry Research	18	483	3.8
Journal of Membrane Science	15	1030	8.4
Journal of CO_2_ Utilization	14	454	7.2
Energy & Fuels	13	503	5.2
ACS Applied Materials & Interfaces	12	390	8.3
Chemosphere	10	123	8.1
Journal of Cleaner Production	9	402	9.7
Carbon Capture Science & Technology	5	70	10.4

**Table 3:** Top 10 most cited articles on CO_2_ capture in Scopus and WoS.

Most Relevant Articles in the Field of CO_2_ Capture	Journal	Cite	Impact Factor (Year 2023)	Ref.
Direct Capture of CO_2_ from Ambient Air	Chemical Reviews	1580	51.4	[32]
Separation and Capture of CO_2_ from Large Stationary Sources and Sequestration in Geological Formations—Coalbeds and Deep Saline Aquifers	Journal of the Air & Waste Management Association	714	2.8	[35]
Recent Advances in Aerogels for Environmental Remediation Applications: A review	Chemical Engineering Journal	551	13.3	[33]
Biopolymer Aerogels and Foams: Chemistry, Properties, and Applications	Journal of the German Chemical Society	538	16.1	[36]
A review of the Hydrate-Based gas Separation (HBGS) Process for Carbon Dioxide Pre-combustion Capture	Energy	510	9.0	[34]
Polymeric Membranes for CO_2_ Separation and Capture	Journal of Membrane Science	298	8.4	[37]
CO_2_-Responsive Polymers	Macromolecular Rapid Communications	244	5.734	[38]
Tunable Polyaniline-Based Porous Carbon with Ultrahigh Surface Area for CO_2_ Capture at Elevated Pressure	Advanced Energy Materials	133	24.4	[39]
Recent Advances in CO_2_-responsive Materials in Separations	Journal of CO_2_ Utilization	72	7.2	[40]
Immobilization of Carbonic Anhydrase on Carboxyl-functionalized Ferroferric Oxide for CO_2_ Capture	International Journal of Biological Macromolecules	26	7.7	[41]

## Data Availability

The data presented in this study are openly available in the article.
